# Crystallization of lysozyme with (*R*)-, (*S*)- and (*RS*)-2-methyl-2,4-pentanediol

**DOI:** 10.1107/S1399004714025061

**Published:** 2015-02-26

**Authors:** Mark Stauber, Jean Jakoncic, Jacob Berger, Jerome M. Karp, Ariel Axelbaum, Dahniel Sastow, Sergey V. Buldyrev, Bruce J. Hrnjez, Neer Asherie

**Affiliations:** a Department of Physics, Yeshiva University, 2495 Amsterdam Avenue, New York, NY 10033-3312, USA; b Department of Biology, Yeshiva University, 2495 Amsterdam Avenue, New York, NY 10033-3312, USA; c National Synchrotron Light Source, Brookhaven National Laboratory, Building 725D, Upton, NY 11973-5000, USA; d Collegiate School, 260 West 78th Street, New York, NY 10024-6559, USA

**Keywords:** MPD, crystallization additives, precipitants, high-resolution protein structures, chirality

## Abstract

Chiral control of crystallization has ample precedent in the small-molecule world, but relatively little is known about the role of chirality in protein crystallization. In this study, lysozyme was crystallized in the presence of the chiral additive 2-methyl-2,4-pentanediol (MPD) separately using the *R* and *S* enantiomers as well as with a racemic *RS* mixture. Crystals grown with (*R*)-MPD had the most order and produced the highest resolution protein structures. This result is consistent with the observation that in the crystals grown with (*R*)-MPD and (*RS*)-MPD the crystal contacts are made by (*R*)-MPD, demonstrating that there is preferential interaction between lysozyme and this enantiomer. These findings suggest that chiral interactions are important in protein crystallization.

## Abstract 2

Crystallization of lysozyme with (*R*)-2-methyl-2,4-pentanediol produces more ordered crystals and a higher resolution protein structure than crystallization with (*S*)-2-methyl-2,4-pentanediol. The results suggest that chiral interactions with chiral additives are important in protein crystal formation.

## 1. Introduction   

Proteins are difficult to crystallize. According to the most recently available statistics from the Structural Biology Knowledgebase (Gabanyi *et al.*, 2011 ▶), fewer than one in eight purified proteins produces diffraction-quality crystals. Furthermore, this success rate has been decreasing over the past decade (Chayen, 2002 ▶, 2004 ▶; Chayen & Saridakis, 2008 ▶); one explanation offered is that the proteins which are easy to crystallize were tackled first (Pusey *et al.*, 2005 ▶).

A protein will crystallize when the solution conditions are thermodynamically and kinetically favorable (Candoni *et al.*, 2012 ▶). As there is currently no way to predict these favorable conditions, protein crystallization remains essentially a brute-force endeavor: many different conditions are examined in the hope that at least one of them will produce crystals (Chan *et al.*, 2013 ▶; Wilson & DeLucas, 2014 ▶). One common way to alter the solution conditions is through the use of additives. These additives are typically salts, small organic molecules and polymers (Dumetz *et al.*, 2009 ▶; McPherson *et al.*, 2011 ▶), although other additives have been used, such as silicon-based surfaces that promote nucleation (Chayen *et al.*, 2001 ▶; Ghatak & Ghatak, 2011 ▶; Tsekova *et al.*, 2012 ▶).

Much has been carried out to understand the role of protein–additive interactions in crystal formation (McPherson, 1999 ▶). While the detailed mechanisms through which additives promote protein crystallization are often not known, a few general features of protein–additive interactions are understood. For example, additives can form favorable crystal contacts leading to a stable and highly ordered crystalline arrangement of proteins (McPherson *et al.*, 2011 ▶).

We are investigating an aspect of protein–additive inter­actions that is relatively unexplored in the context of protein crystallization: chirality. Chiral control of crystallization has ample precedent in the small-molecule world (Addadi *et al.*, 1982 ▶; Amharar *et al.*, 2012 ▶; Blackmond, 2011 ▶; Brittain, 2013 ▶; Eicke *et al.*, 2013 ▶; Gou *et al.*, 2012 ▶; Levilain *et al.*, 2012 ▶; Lorenz & Seidel-Morgenstern, 2014 ▶), but most of the work on chiral interactions between proteins and additives has focused on how such interactions control protein function (Brooks *et al.*, 2011 ▶). Typically, when a protein binds a small chiral molecule, it interacts differently with one enantiomer than with the other because the protein itself is chiral. In principle, such chiral interactions may affect not only protein function but also protein phase behaviour, including crystallization.

Our previous work on thaumatin and sodium tartrate demonstrated that the chirality of the additive has a substantial effect on the habit, packing, solubility and growth of protein crystals (Asherie, Ginsberg, Blass *et al.*, 2008 ▶; Asherie, Ginsberg, Greenbaum *et al.*, 2008 ▶; Asherie *et al.*, 2009 ▶). Furthermore, by working with enantiomerically pure additives, we were able to determine the highest resolution (0.94 Å) thaumatin structure currently available (Asherie *et al.*, 2009 ▶).

To examine the generality of our findings, we are studying other pairs of proteins and chiral precipitants. Here, we discuss our results for the crystallization of lysozyme with 2-methyl-2,4-pentanediol (MPD; C_6_H_14_O_2_). We chose this protein–precipitant pair for three reasons. Firstly, lysozyme is the most widely examined protein in crystallization and structural analysis studies (Chayen & Saridakis, 2001 ▶; Liang *et al.*, 2013 ▶; Magay & Yoon, 2011 ▶; Tu *et al.*, 2014 ▶). Secondly, MPD is a chiral molecule that is one of the most common additives in protein crystallization (Anand *et al.*, 2002 ▶), although it has been used exclusively as the racemate. Thirdly, lysozyme has previously been crystallized with (*RS*)-MPD, but the results of these investigations are contradictory: Weiss and coworkers found only (*R*)-MPD in the crystal (Weiss *et al.*, 2000 ▶), whereas Michaux and coworkers found only (*S*)-MPD (Michaux *et al.*, 2008 ▶).

In the current study, we crystallized lysozyme using the individual *R* and *S* enantiomers of MPD separately as well as the racemate, and determined the X-ray structures of the resultant crystals. We also determined the X-ray structure of lysozyme crystals grown without MPD. All structures were obtained to high resolution (1.25 Å or better), allowing a detailed comparison of the protein structures and, in principle, an unambiguous assignment of the absolute configuration (*R* or *S*) of the MPD molecules. This assignment, however, is complicated by the fact that the MPD molecule can adopt different conformations (Anand *et al.*, 2002 ▶). We therefore performed a detailed conformational analysis of MPD using quantum-chemical (QC) calculations and molecular-dynamics (MD) simulations. Consequently, we were able to use the stable conformer that is likely to dominate the relative conformer population in our analysis of the protein crystal structures. Ambiguity in the analysis is thereby diminished.

We find that crystals grown with (*R*)-MPD had the least disorder (as measured by the mosaicity and *B* factor) and produced the highest resolution protein structures. This finding is consistent with the observation that co-crystallization with either (*R*)- or (*RS*)-MPD gives crystal contacts made exclusively by (*R*)-MPD, demonstrating that there is preferential interaction between lysozyme and this enantiomer. These results support the hypothesis that chiral interactions may be important in protein crystallization with chiral additives.

## 2. Materials and methods   

### 2.1. Materials   

Lysozyme (catalog No. 2933, lot No. 36P9210) was purchased from Worthington Biochemical Corporation, Lakewood, New Jersey, USA. (*R*)-MPD and (*S*)-MPD were synthesized by Reuter Chemische Apparatebau KG, Freiburg, Germany. (*RS*)-MPD (catalog No. 68340, lot No. 1345630) was purchased from Sigma–Aldrich, St Louis, Missouri, USA. Tris base (catalog No. BP512-500), sodium azide (catalog No. S227I-500) and hydrochloric acid (catalog No. A144S-500) were purchased from Fisher Scientific, Pittsburgh, Pennsylvania, USA. All materials were used without further purification. The purity of the protein and the chemical and enantiomeric purity of (*R*)-, (*S*)- and (*RS*)-MPD were determined as described in the Supporting Information. Deionized water was obtained from an Integral 3 deionization system (Millipore, Billerica, Massachusetts, USA). Solutions were filtered through a Nalgene disposable 0.22 µm filter unit (Nalge Nunc International, Rochester, New York, USA) prior to use.

Concentration measurements were carried out by UV–Vis extinction spectroscopy on a Beckman–Coulter DU800 spectrophotometer. The extinction coefficient of lysozyme at 280 nm was taken to be ∊^0.1%^ = 2.64 mg ml^−1^ cm^−1^ (Aune & Tanford, 1969 ▶). Conductivity and pH measurements were performed using an Orion 4-Star conductivity and pH meter with a DuraProbe conductivity cell and a ROSS Sure-Flow pH electrode (Thermo Fisher Scientific, Waltham, Massachusetts, USA).

### 2.2. Protein crystallization   

Lysozyme was dissolved in 200 m*M* Tris (titrated to pH 8.0 with HCl; σ = 9.90 mS cm^−1^), washed three times in the same buffer in an Amicon Ultra-4 centrifugal filter device with a 3 kDa molecular-weight cutoff (Millipore, Billerica, Massachusetts, USA) and then concentrated to approximately 35 mg ml^−1^. Crystals were grown using the hanging-drop vapor-diffusion method in the EasyXtal 15-Well Tool (catalog No. 132006; Qiagen, Valencia, California, USA). Drops were made by mixing 10 µl protein solution with 10 µl reservoir solution. This mixture was vortexed briefly and three 5 µl drops were then dispensed onto the crystallization supports. The reservoir solutions (300 µl) were 60%(*v*/*v*) (*R*)-, (*S*)- or (*RS*)-MPD in water. A control experiment with only water in the reservoir was also carried out. The crystallization trays were left at 4.0 ± 0.5°C and inspected periodically by bright-field microscopy with an AxioImager A1m microscope (Carl Zeiss, Göttingen, Germany). Crystals of roughly 200 µm in size grew in about 5 d with MPD (Supplementary Fig. S1); crystals of similar size took about two weeks to grow in the control. The crystals were harvested with mounted cryoloops (Hampton Research, Aliso Viejo, California, USA). No cryoprotectant was used, except for the crystals grown in the control, which were dipped into Paratone N (Hampton Research, Aliso Viejo, California, USA) immediately before the diffraction measurements.

### 2.3. Data collection, refinement and structural analysis   

All X-ray diffraction data were recorded on beamline X6A at the National Synchrotron Light Source, Brookhaven National Laboratory, Upton, New York, USA between 13.5 and 15.1 keV. All data were recorded at 100 K using an ADSC Q270 CCD detector (ADCS, Poway, California, USA). Data were indexed, integrated and scaled in *HKL*-2000 (Otwinowski & Minor, 1997 ▶). Lysozyme crystal structures were solved by molecular replacement using *MOLREP* (Vagin & Teplyakov, 2010 ▶) and the model from PDB entry 1iee (Sauter *et al.*, 2001 ▶). Each model was refined by restrained maximum-likelihood refinement with *REFMAC* (Murshudov *et al.*, 2011 ▶; Winn *et al.*, 2011 ▶) with individual anisotropic temperature factors and manual building performed in *Coot* (Emsley *et al.*, 2010 ▶). After the final refinement, the stereochemistry of the structures was assessed with *PROCHECK* (Laskowski *et al.*, 1993 ▶). All figures were prepared with *PyMOL* (http://www.pymol.org).

### 2.4. Quantum-chemical calculations and molecular-dynamics simulations   

*Ab initio* electronic structure calculations for the nine (*R*)-MPD conformers **1**–**3** (see Fig. 2) were performed with *Gaussian*09 (Frisch *et al.*, 2009 ▶). Stationary points were located on the local potential surfaces for these nine conformers at a modest level of quantum-chemical theory, using the second-order Møller–Plesset perturbation (MP2) method (Møller & Plesset, 1934 ▶) and the 6-311++G(d,p) medium-sized basis (Clark *et al.*, 1983 ▶) set for C, H and O atoms. The program package defaults, including criteria for wavefunction convergence and locations of stationary points on potential surfaces, were used. The nine initial input structures were the result of a qualitative conformational analysis of the all-staggered conformational possibilities for (*R*)-MPD. Conformer **1a** was assumed to be intramolecularly hydrogen-bonded; we were able to focus on a single conformer because the geometry-optimized energies of different intramolecularly hydrogen-bonded rotamers about C—O bonds differed by less than 1 kJ mol^−1^. As expected, inversion of configuration at C4 to give (*S*)-MPD gave identical computational results.

We examined the effect of water solvation on the relative conformer energies at the same level of quantum-chemical theory, incorporating the default integral equation formalism variant (IEF) within the polarizable continuum model (PCM) for placing a solute in a cavity within the solvent reaction field (SCRF; Tomasi *et al.*, 2005 ▶).

Molecular-dynamics simulations of both (*R*)-MPD and (*S*)-MPD were performed using *GROMACS* v.4.0.5 (Hess *et al.*, 2008 ▶). The initial coordinates for (*R*)- and (*S*)-MPD were taken from PDB entries 4b4e and 4b4i, respectively, with H atoms added using the *pdb*2*gmx* utility of *GROMACS*. Simulations were run *in vacuo* with one molecule of either (*R*)- or (*S*)-MPD placed in a cubic box of length 3.0 nm with periodic boundary conditions. Since MPD has no net charge, no counterions were added. The topology files were constructed using parameters for OPLS-AA atom types (Jorgensen *et al.*, 1996 ▶; Jorgensen & Tirado-Rives, 1988 ▶). The properties of the atoms used in the topology file for both enantiomers are shown in Supplementary Table S1.

The OPLS-AA all-atom force field was used in running simulations. Each simulation box was subjected to energy minimization using the steepest-descent method. Simulations were run using the NVT ensemble, and the temperature was held constant at 300 or 370 K using the V-rescale thermostat (Bussi *et al.*, 2009 ▶) with a coupling constant of 0.1. Electrostatic interactions were treated using the particle mesh Ewald algorithm (Essmann *et al.*, 1995 ▶) using electrostatic, van der Waals and neighbor-list cutoffs of 0.9 nm. The *SHAKE* constraint algorithm (Ryckaert *et al.*, 1977 ▶) was used to constrain all bonds with a tolerance of 0.0002. Simulations were run for 100 ns, using 1 fs time steps and saving coordinates and energies every 1 ps. The first 10 ns of each simulation were considered to be equilibration time and were not used in subsequent analysis.

Annealing simulations were run with (*R*)-MPD *in vacuo*. The systems were prepared as above. In each of 100 simulations, the initial velocities were independently randomly generated. The initial temperature was 370 K and the simulation was run for 200 ps. Over every subsequent 200 ps, the temperature was decreased linearly with time by 5 K. Thus, 14.6 ns after the beginning the simulations the temperature reached 5 K. During the next 200 ps, the temperature was decreased linearly with time to 0.1 K. The simulation continued at this temperature until it had run for a total of 20 ns.

Trajectory analysis was performed with the *GROMACS* utilities package. For clarity, we report torsion angles in the range 0 to 360° instead of the customary −180 to 180° (torsion angles greater than 180° can be converted to the usual negative torsion angles by subtracting 360°).

### 2.5. Database analysis   

MPD conformations were extracted from the RCSB Protein Data Bank (PDB; http://www.pdb.org; Berman *et al.*, 2000 ▶) and the Cambridge Structural Database (CSD; Allen, 2002 ▶). The PDB was searched using the chemical IDs for either (*R*)-MPD (MRD) or (*S*)-MPD (MPD) together with two additional requirements: X-ray resolution between 0 and 1.5 Å and sequence homology of less than 90% to other macromolecules. This search yielded 49 protein structure hits for MRD and 89 protein structure hits for MPD. Some of these hits contained both enantiomers, yielding 117 unique protein structures with (*R*)- or (*S*)-MPD. [We note that numerous protein structures had more than one (*R*)- or (*S*)-MPD molecule associated with them.] These molecules were inspected using *Coot* (Emsley *et al.*, 2010 ▶) with the structure and electron-density maps (2*F*_o_ − *F*_c_ and *F*_o_ − *F*_c_) downloaded through the Uppsala Electron Density Server (Kleywegt *et al.*, 2004 ▶). Hits that had no density or no structure factors were discarded. The quality of the electron-density map, the local hydrogen bonding and the atomic *B* factors of the molecules were used to check whether the assigned model (*R*)- or (*S*)-MPD structure was acceptable as is, *i.e.* whether the enantiomer and conformer selected were supported by the data. Acceptable structures were kept, while unacceptable structures were either discarded (because the torsion angles of the molecule could not be determined unambiguously) or reassigned to achieve a better agreement between the model and the electron density. The torsion angles of the acceptable and reassigned structures were measured using the built-in function of *Coot*. A total of 221 molecules were retained: 109 were (*R*)-MPD and 112 were (*S*)-MPD.

The CSD was searched with *ConQuest* (Bruno *et al.*, 2002 ▶) using the chemical formula of MPD (C_6_H_14_O_2_). The resulting 28 hits were inspected and only the seven hits that corresponded to 2-methyl-2,4-pentanediol were retained (reference codes BACXIM10, FALDUS, KOFPAW, NIRQIO, NOSVOG, PIVYEZ and TECYIJ). The hits, some of which contained multiple molecules of both enantiomers, were examined using *Mercury* (Bruno *et al.*, 2002 ▶). Since no structure-factor information was available, the assigned model (*R*)-MPD or (*S*)-MPD structures were accepted as is, unless the local hydrogen bonding suggested that the structure be reassigned. The torsion angles of the acceptable and reassigned structures were measured using the built-in function of *Mercury*. A total of ten molecules were retained: three were (*R*)-MPD and seven were (*S*)-MPD.

## 3. Results and discussion   

### 3.1. Protein and precipitant purity   

Protein purity is a crucial factor in crystallization (McPherson, 1999 ▶). Indeed, the deleterious effect of impurities on the crystallization of lysozyme has been studied extensively (Dold *et al.*, 2006 ▶; Judge *et al.*, 1998 ▶; Lorber *et al.*, 1993 ▶; Parmar *et al.*, 2007 ▶; Thomas *et al.*, 1996 ▶). We chose to work with lysozyme from Worthington Biochemical Corporation because it has been shown to produce high-quality crystals (Parmar *et al.*, 2007 ▶). Our characterization of the protein by size-exclusion and cation-exchange high-performance chromatography, quasi-elastic light scattering and electrospray ionization mass spectroscopy confirmed its high purity (Supplementary Figs. S2 and S3 and Table S2).

For the precipitant, both chemical and enantiomeric purity must be controlled. We purchased (*RS*)-MPD from Sigma–Aldrich, but decided against using the commercially available (*R*)-MPD from the same manufacturer (catalog No. 252840) because of its lower chemical purity and the limited information about its enantiomeric purity (only the specific rotation is given). For (*S*)-MPD, a commercial supplier was not an option. To our knowledge, this enantiomer is not available as a common chemical.

**Figure 1:**
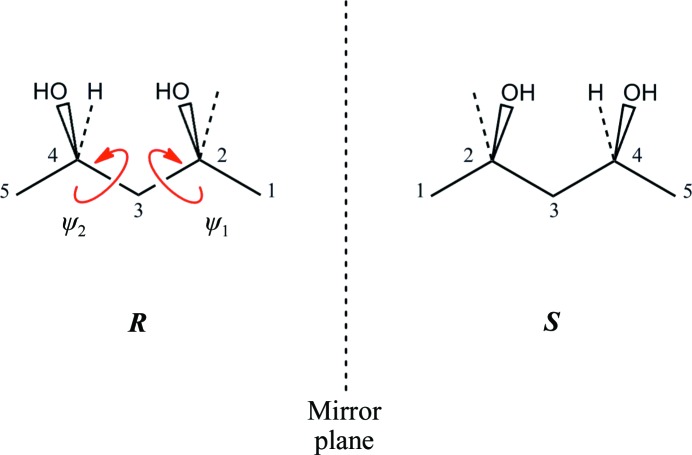
Schematic representation of (*R*)- and (*S*)-MPD. The C atoms are numbered according to the convention used in this work and the torsion angles (ψ_1_, ψ_2_) are represented by red arrows. CM is the unlabelled methyl C atom attached to C2 by a dashed line; the O atoms attached to C2 and C4 are O2 and O4, respectively, and the chiral center is at C4.

We therefore commissioned the custom syntheses of (*R*)-MPD and (*S*)-MPD by Reuter Chemische Apparatebau KG (the structures of the two enantiomers and the associated nomenclature are shown in Fig. 1 ▶). Since (*S*)-MPD has not been characterized previously and only partial information is available for (*R*)-MPD, we analyzed these enantiomers and the racemate (*RS*)-MPD by ^1^H and ^13^C NMR, tandem mass spectrometry and optical activity (Supplementary Figs. S4–S9 and Tables S3 and S4). For completeness, we also provide the gas-chromatography results of (*R*)-MPD and (*S*)-MPD that were given to us by the manufacturer (Supplementary Figs. S10 and S11). These results confirm the chemical identity of the molecules synthesized and demonstrate their high chemical and enantiomeric purity; the key results are summarized in Table 1 ▶.

### 3.2. MPD conformation   

An important step in the analysis of the X-ray diffraction data is the proper assignment of any MPD molecules that are present in the crystal structure. This assignment involves selecting the enantiomer(s) and conformer(s) of the molecule that best fit the electron density. For the experiments with pure (*R*)- or (*S*)-MPD there is only one enantiomer to choose, but for crystallization with (*RS*)-MPD the selection is less straightforward.

If the electron-density map is of sufficiently high quality, it is possible to distinguish the two enantiomers by inspecting the shape of the map, even though the H atom on the chiral center C4 (Fig. 1 ▶) is not visible in the X-ray data. An example is the (*R*)-MPD molecule found near Phe34 by Weiss and coworkers in the structure of lysozyme (PDB entry 1dpw) crystallized with (*RS*)-MPD (Weiss *et al.*, 2000 ▶). If the shape of the map is inconclusive, knowledge of the most likely conformer can be helpful in selecting the appropriate enantiomer.

Since H atoms contribute little to the electron density, the conformation of MPD as obtained from the electron-density map is completely determined by the torsion angles (ψ_1_, ψ_2_), which are defined by C atoms C1—C2—C3—C4 and C2—C3—C4—C5, respectively (Fig. 1 ▶). Furthermore, for an isolated molecule the stable conformers of one enantiomer will be mirror images of the other. Energetic considerations suggest that the expected values of these angles for an isolated (*R*)-MPD molecule are approximately (180°, 180°). This conformation (shown in Fig. 1 ▶) allows the formation of an intramolecular hydrogen bond (the distance between O2 and O4 atoms is 2.8 Å) and corresponds to a favorable arrangement of the C1—C2—C3—C4—C5 backbone (Salam & Deleuze, 2002 ▶). To verify these considerations, we carried out both quantum-chemical calculations and molecular-dynamics simulations on MPD.

**Figure 2:**
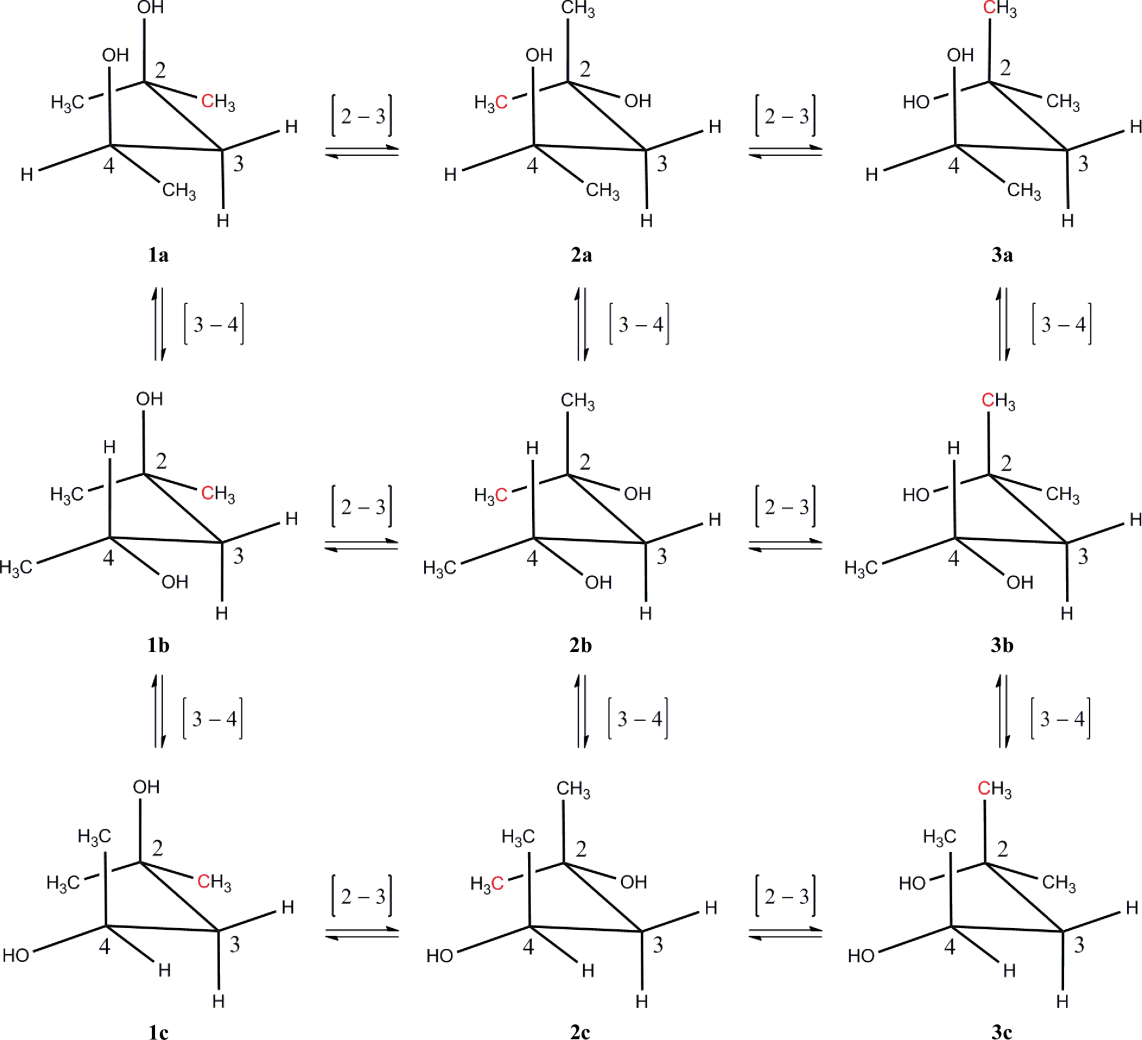
The conformers of MPD studied by quantum-chemical calculations. Each conformer is denoted by a two-symbol code (number and letter) that approximately represents the torsion angles (ψ_1_, ψ_2_) of the initial conformation used in the calculation. The numbers **1**, **2** and **3** correspond to ψ_1_ = 180, 300 and 60°, respectively; the letters **a**, **b** and **c** correspond to ψ_2_ = 180, 60 and 300°, respectively. [For example, **1a** is the (180°, 180°) conformer shown in Fig. 1 ▶.] To interconvert two adjacent structures, a rotation is performed about the carbon–carbon bond given in square brackets. For clarity, only C2, C3 and C4 are numbered; C1 is shown in red, while CM and C5 (which is adjacent to C4) are shown in black.

We performed quantum-chemical (QC) calculations to determine the relative energies of the nine conformers of (*R*)-MPD shown schematically in Fig. 2 ▶. These all-staggered conformers were chosen as the initial configurations for geometry optimization; each of these is likely to be close to a local minimum on the conformational potential energy surface (Mo, 2010 ▶). The relative energies of the optimized geometries for the nine conformers are listed in Table 2 ▶ (see also Supplementary Table S5). As expected, **1a** is the most stable conformer *in vacuo* and the torsion angles of the final, geometry-optimized structure are (177°, 173°), which are close to the qualitatively predicted (180°, 180°). Furthermore, there is a significant gap in energy between **1a** and the next most stable conformer, **3a** (Fig. 2 ▶). Conformer **3a** cannot accommodate an intramolecular hydrogen bond because O2 and O4 are too far apart (3.9 Å). Indeed, the energy difference (12.4 kJ mol^−1^) between **3a** and **1a** falls within the range of hydrogen-bond energies (9.2–24.3 kJ mol^−1^) calculated for other alkanediols (Mandado *et al.*, 2006 ▶). Finally, we verified for conformer **1a** that the (*R*)- and (*S*)-MPD enantiomers have identical energies.

Since our protein crystals form in the presence of solvent, we also calculated the relative energies of the conformers using a polarizable continuum model of water (Scalmani & Frisch, 2010 ▶). While the exact values of the relative energies are slightly different from those found *in vacuo*, the ranking of conformers in terms of stability is the same (Table 2 ▶). In particular, the most stable conformer is **1a** and there is an energy difference corresponding to the loss of the intramolecular hydrogen bond for the next most stable conformer **3a**.

**Figure 3:**
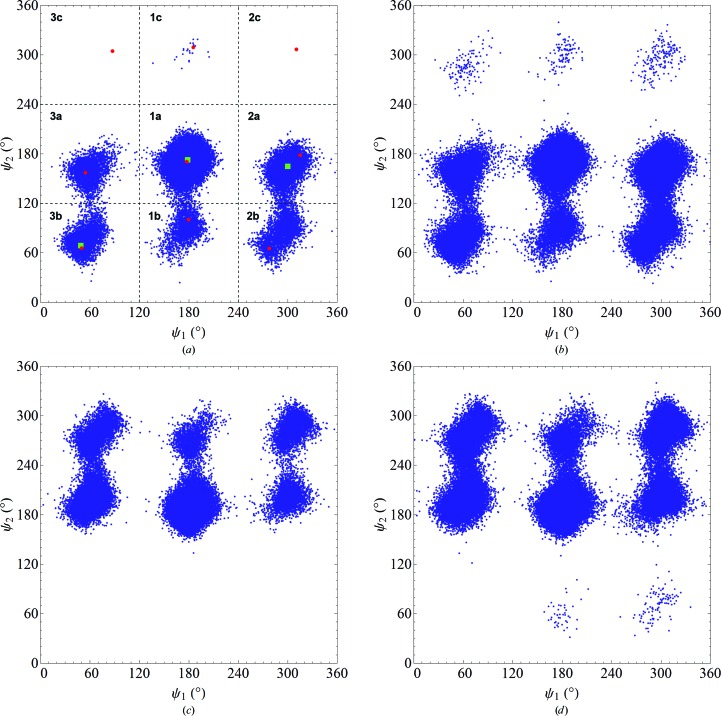
The conformations of MPD from MD simulations *in vacuo*. (*a*) (*R*)-MPD at 300 K; (*b*) (*R*)-MPD at 370 K; (*c*) (*S*)-MPD at 300 K; (*d*) (*S*)-MPD at 370 K. In (*a*) the dashed lines mark the nine bins used in the free-energy calculations. The red circles are the nine locally stable conformers obtained from quantum-chemical calculations; the corresponding label for each conformer is shown in bold. The results of the simulated-annealing experiments lie within the green squares.

The results above indicate that **1a** is the most stable conformer in a variety of environments. However, the actual population of conformers in any given environment will not solely be determined by energetic factors, but also by entropic factors, *i.e.* by the relative free energies. To determine the relative free energies of MPD, we performed MD simulations *in vacuo* at 300 and 370 K for each enantiomer. The (ψ_1_, ψ_2_) conformations recorded every 1 ps during the 100 ns simulations are shown in Fig. 3 ▶. (The first 10 ns of each simulation were taken to be equilibration time and were not included in our analysis.) We see that the data cluster around the conformers examined using QC calculations (red circles in Fig. 3 ▶ *a*). At 300 K not all of the conformers are accessible to the MD simulations, but at 370 K all nine conformers are observed for (*R*)-MPD and only one is not observed for (*S*)-MPD. Furthermore, no extraneous conformers are found, confirming that our choice of conformers for the QC calculations was reasonable. Finally, at each temperature the (*R*)- and (*S*)-MPD results show approximately the expected mirror symmetries. More specifically, for the null hypothesis that the underlying distributions of the (*R*) and (*S*) conformers are the same, a χ^2^ test (see Supporting Information §S2) reveals that this hypothesis is accepted with *p*-values of greater than 0.90 (at 300 K) or 0.80 (at 370 K).

The relative free energy of each conformer was calculated by dividing the (ψ_1_, ψ_2_) conformational space into nine equal-sized bins (Fig. 3 ▶ *a*). Since only one conformer lies in each bin, all observations in a given bin can be associated with a specific conformer. The molar Helmholtz free energy Δ*F_i_* of conformer *i* relative to **1a** is given by (Frenkel & Smit, 1996 ▶)
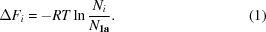
Here, *N_i_* is the number of observation of conformer *i* and *N*_**1a**_ is the number of observations of conformer **1a**. *R* is the universal gas constant and *T* is the absolute temperature.

As we found with our QC calculations, the MD simulations reveal that **1a** is the most stable conformer and that there is a gap to the next most stable conformer (Table 3 ▶). When the temperature is changed from 300 to 370 K this gap decreases, illustrating the increased importance of entropic contributions. Indeed, at 370 K the order of relative stability of the conformers is not the same as that at 300 K.

Although the energy (QC) and free-energy (MD) landscapes share global features, they differ in several ways. For example, the next most stable conformer in terms of energy is **3a**, while in terms of free energy it is **2a**. (We note that **2a** is the third most stable conformer energetically, only 1.4 kJ mol^−1^ higher than **3a** *in vacuo*; Table 2 ▶.) Also, according to the MD results, **1c**, **2c** and **3c** are the least stable states and have similar free energies at 370 K, but the QC calculations show that **3c** is energetically more stable than the other two by about 10 kJ mol^−1^, which is approximately the energy of a hydrogen bond. In fact, the distance between the O2 and O4 atoms in **3c** is 2.7 Å, which is suitable for the formation of a strong hydrogen bond. It is likely that the inclusion of entropic effects in the MD simulation renders **3c** less stable than would be predicted on purely energetic considerations.

To further compare the QC and MD results, we ran 100 MD annealing simulations on (*R*)-MPD in which the system was equilibrated at 370 K, where all nine conformers are observed, and slowly cooled to 0.1 K, where entropic contributions are small and energetic considerations determine the stability of the molecule. In 77 of these runs we found that the final conformation (green square in the central bin of Fig. 3 ▶ *a*) coincided with the **1a** conformation of the QC calculations (red circle in the central bin of Fig. 3 ▶ *a*), which is consistent with our previous result that **1a** is a global energy minimum.

In 20 of the remaining 23 runs, (*R*)-MPD reached a local minimum in the **2a** bin (the next most stable state in the MD simulations after **1a**; Table 3 ▶), while in three runs it reached the **3b** conformation (the third most stable state). Based on the QC results we would have expected the order of states in the low-temperature limit to be **1a** < **3a** < **2a** (in terms of increasing energy; Table 2 ▶). The difference may reflect the limitations of a classical force field in capturing the quantum-mechanical aspects of atomic interactions.

**Figure 4:**
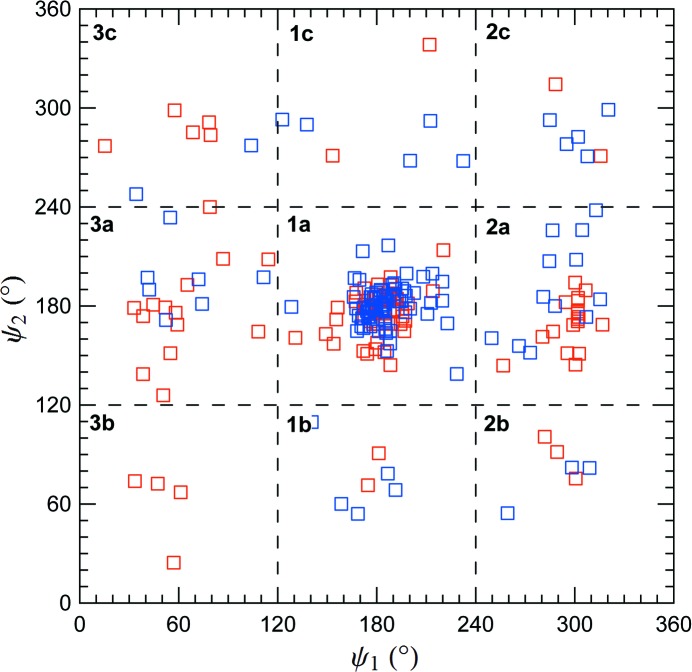
The conformations of MPD from the PDB. The two enantiomers are shown in different colors: (*R*)-MPD in red and (*S*)-MPD in blue. The dashed lines mark the nine bins used in the free-energy calculations (*cf*. Fig. 3 ▶ *a*).

Our computational results strongly suggest that **1a** should be the conformer that is most frequently observed in nature. To verify this hypothesis, we examined the (ψ_1_, ψ_2_) conformations of (*R*)- and (*S*)-MPD found in the PDB (Berman *et al.*, 2000 ▶). As expected, we observed that majority of the conformers cluster around **1a** (Fig. 4 ▶), in agreement with a similar analysis made about a decade ago on the MPD molecules available at the time (Anand *et al.*, 2002 ▶). To further examine the MPD–protein interactions, we binned the PDB data as we did for the MD results and calculated the relative Gibbs free energies of the conformers (Table 4 ▶). The results follow the pattern of the QC calculations and MD simulations in the absence of protein: conformer **1a** is the most stable and there is a gap (3.7 kJ mol^−1^ at 300 K) between **1a** and the next most stable conformer. For the PDB results this next most stable conformer is **2a**, which in the same result as we found in the MD simulations (Table 3 ▶).

In addition to providing information about the relative stability of the MPD conformers when strongly associated with protein, the PDB results allow us to examine possible chiral effects on this relative stability. In particular, we can ask whether there is a statistically significant difference in the distribution of conformers for co-crystallized (*R*)- and (*S*)-MPD. Since the MPD molecules in the PDB database are found in a chiral environment, a difference in the conformer distributions is possible. A χ^2^ test (Press *et al.*, 1992 ▶) on the binned data of the (*R*)- and (*S*)-MPD distributions (*i.e.* the number of times that each of the nine conformers is observed) suggests that the null hypothesis, *i.e.* that the underlying distributions of the (*R*) and (*S*) conformers are the same, should be accepted (*p* = 0.11). This result supports the hypothesis that any chiral interaction between MPD and proteins does not affect the relative stability of the conformers.

As a further check on the conformations of MPD, we also examined the Cambridge Structural Database (CSD; Allen, 2002 ▶). While the small sample size precluded a detailed statistical analysis, we note that eight of the ten MPD molecules extracted from the CSD adopt conformer **1a**.

The QC, MD and database results all indicate that **1a** is the most stable conformer of MPD in both chiral and achiral environments. The other eight conformers are significantly less stable; the database analysis reveals that the probability of finding the MPD molecule in a conformation other than **1a** is less than 50%, and the simulation results give much lower probabilities. We therefore decided to use **1a** exclusively to fit our electron-density data for MPD co-crystallized with protein. As we show in §[Sec sec3.3]3.3, this choice leads to a reasonable fit of the experimental data.

Given the high resolution of our X-ray structures, we could have added one or even two more conformers with low occupancy to slightly improve the fit for the MPD molecules. However, we chose a conservative approach to the interpretation of the electron-density map in order to achieve physically meaningful results and to minimize the chance of encountering the many problems that can arise when analyzing protein–ligand complexes (Kleywegt, 2009 ▶). Our work highlights the need for a thorough conformational analysis of the ligand molecules complexed with macromolecular structures, and we support the recent appeal for reliable standard restraint libraries for ligand molecules found in the PDB (Jaskolski, 2013 ▶).

### 3.3. Lysozyme crystals and MPD interactions   

When lysozyme is crystallized in Tris at pH 8.0, it forms tetragonal crystals with space group *P*4_3_2_1_2 and almost identical unit-cell parameters whether or not MPD is used and independent of the MPD stereochemistry (Table 5 ▶). This result is consistent with the findings of other investigators, who have observed this crystalline arrangement for a broad set of solution conditions (Bujacz *et al.*, 2010 ▶; Helliwell & Tanley, 2013 ▶; Judge *et al.*, 1999 ▶; Michaux *et al.*, 2008 ▶; Tanley *et al.*, 2012 ▶; Weiss *et al.*, 2000 ▶). The structures of lysozyme obtained from the four conditions that we have studied [namely (*R*)-, (*S*)-, (*RS*)-MPD and no MPD] are similar as well; the root-mean-square deviation between equivalent C^α^ atoms is less than 0.4 Å for any two of the structures (Supplementary Table S6).

**Figure 5:**
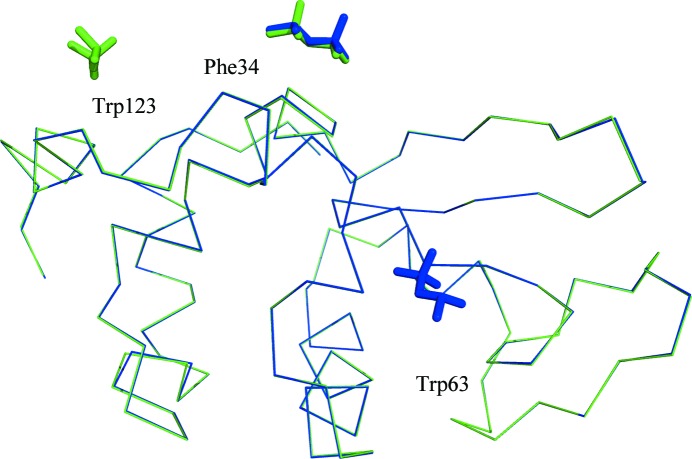
Overall structures of lysozyme with the enantiomers of MPD. A ribbon diagram of the C^α^ backbone is shown for crystals grown with (*R*)-MPD (blue) and (*S*)-MPD (green). The MPD molecules associated with each structure are shown in the same color as the protein backbone.

There are, however, important differences between the crystals. Most importantly, the resolution and disorder (as measured by the mosaicity and *B* factor) vary with the precipitant used (Table 5 ▶). The highest quality crystals are produced when (*R*)-MPD is the precipitant. Furthermore, the pure enantiomers of MPD make different interactions with the protein. Both (*R*)-MPD and (*S*)-MPD form a crystal contact near Phe34, but a second molecule of (*R*)-MPD is found near Trp63 very close to the active site of the protein (Ogata *et al.*, 2013 ▶). No (*S*)-MPD is observed at this location, but a second molecule of (*S*)-MPD is found near Trp123 (Fig. 5 ▶). With (*RS*)-MPD as the precipitant, only (*R*)-MPD molecules are observed and they are found near Phe34 and Trp63, the same pattern as observed for pure (*R*)-MPD. We examine in detail below each of the sites where MPD is found.

#### 3.3.1. Phe34 interaction site   

**Figure 6:**
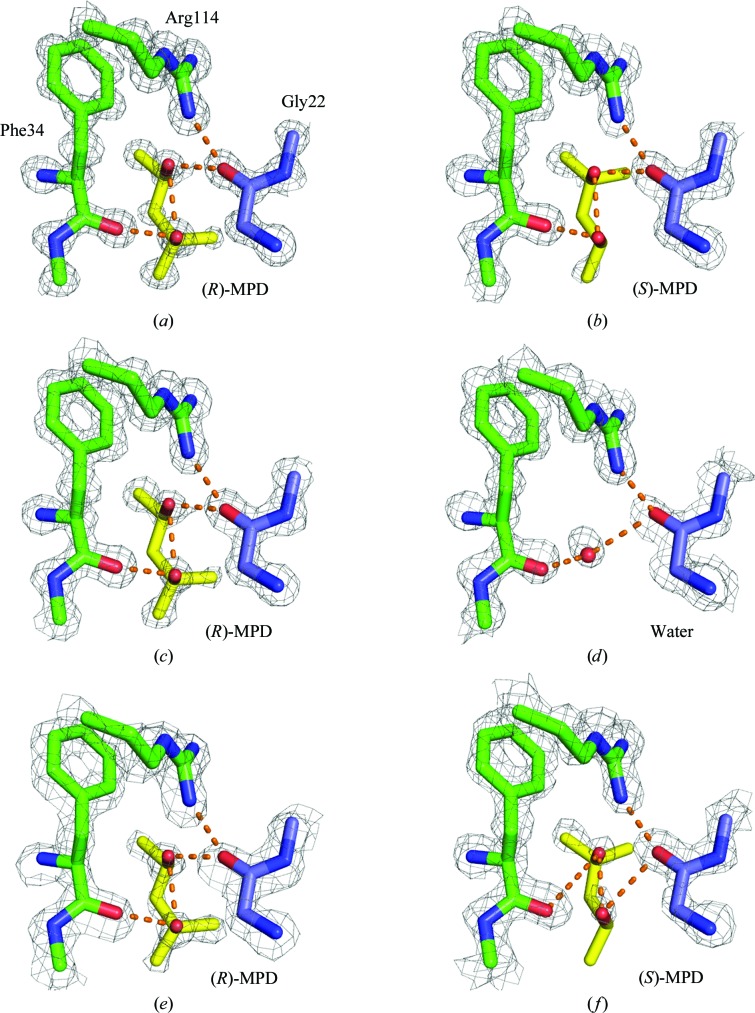
The crystal contact site near Phe34. Two symmetry-related protein molecules (shown with green and purple C atoms) interact through hydrogen bonds (dashed orange lines). (*a*)–(*d*) depict the results from the current work with different additives: (*a*) (*R*)-MPD; (*b*) (*S*)-MPD; (*c*) (*RS*)-MPD; (*d*) no MPD added. The red sphere in (*d*) is the O atom of a water molecule. For comparison, the results of (*e*) Weiss *et al.* (2000 ▶) and (*f*) Michaux *et al.* (2008 ▶), both of which used (*RS*)-MPD, are also presented. The 2*F*_o_ − *F*_c_ density is shown as a gray mesh contoured at 1.5σ.

This is a crystal contact site where MPD forms hydrogen bonds involving residues on two symmetry-related protein molecules: Phe34 and Gly22′ (Fig. 6 ▶; for completeness, the direct hydrogen bond between Arg114 and Gly22′ is also shown). This site has been discussed previously by other investigators working at lower resolution with (*RS*)-MPD, but the results are contradictory: Weiss and coworkers found only (*R*)-MPD in the crystal (Weiss *et al.*, 2000 ▶), while Michaux and coworkers found only (*S*)-MPD (Michaux *et al.*, 2008 ▶).

Our high-resolution structures with pure (*R*)- and (*S*)-MPD allow us to clarify these conflicting results. We observe that the electron density is better defined for (*R*)-MPD (Fig. 6 ▶ *a*) than it is for (*S*)-MPD (Fig. 6 ▶ *b* and Supplementary Fig. S12), which is reflected in the higher occupancy of (*R*)-MPD (0.9 *versus* 0.5). Indeed, we find that a water molecule (occupancy 0.5) at the C1 position of (*S*)-MPD is competing with (*S*)-MPD (for clarity, this water molecule is omitted from Fig. 6 ▶ *b*). These results suggest that there is a preferential interaction between lysozyme and (*R*)-MPD at the Phe34 site, and this suggestion is confirmed by our results with (*RS*)-MPD: we observe (*R*)-MPD at the site (Fig. 6 ▶ *c*). The occupancy (0.55) is not as high as that of pure (*R*)-MPD, which may be owing to several factors, such as the lower concentration of (*R*)-MPD in solution [half of that in pure (*R*)-MPD] and the competition with (*S*)-MPD for the site.

Also, just as we observed with pure (*S*)-MPD, it is possible that water is competing for the same site. When we crystallize lysozyme without any MPD, we observe a water molecule that makes equivalent hydrogen bonds (Fig. 6 ▶ *d*). Nevertheless, MPD is a more effective crystallizing agent than water. Lysozyme crystals (200 µm) grew in about 5 d with MPD; crystals of approximately the same size took about two weeks to grow without MPD. In previous work, we noticed a similar effect with thaumatin and the l and d enantiomers of sodium tartrate, where the same crystal habits form with and without the additives but those grown with the additives are favored kinetically (Asherie *et al.*, 2009 ▶).

Given these results, we agree with the assignment of Weiss *et al.* (2000 ▶) of (*R*)-MPD at the Phe34 site when (*RS*)-MPD is used as the additive (Fig. 6 ▶ *e*). While it is possible that there is a small fraction of (*S*)-MPD contributing to the electron density, assigning only (*S*)-MPD to the site, as performed by Michaux *et al.* (2008 ▶), provides a poor fit to the electron-density data. The problematic nature of their assignment can be seen by examining the *B* factors of the atoms in (*S*)-MPD, which are non-uniform and unusually high (Supplementary Fig. S13). When we produced an OMIT map using their data, we find that assigning (*R*)-MPD to the site provides a better fit to the electron density.

Another difference between the interactions of each enantiomer with the protein is evident from the different orientations of the two additives at the binding site (Figs. 6 ▶ *a* and 6 ▶ *b*), which lead to different intermolecular hydrogen bonding between MPD and the protein residues. The donor–acceptor pairs connecting the (*R*)-MPD and the protein are O2 and the carbonyl O atom of Phe34 and O4 and the carbonyl O atom of Gly22′, whereas for (*S*)-MPD the pairs are O4–Phe34 and O2–Gly22′.

As it is possible for O2 and O4 to make hydrogen bonds to either Phe34 or Gly22′, we wondered why there is no evidence in the electron density for a rigid-body rotation in which O2 and O4 exchange locations. We believe that such a rotation is unfavorable owing to steric hindrance. For example, rotating (*R*)-MPD so that it makes the O4–Phe34 and O2–Gly22′ hydrogen bonds observed for (*S*)-MPD would lead to a strong repulsion between the methyl group CM and the carbonyl O atom of Lys33.

#### 3.3.2. Trp63 interaction site   

**Figure 7:**
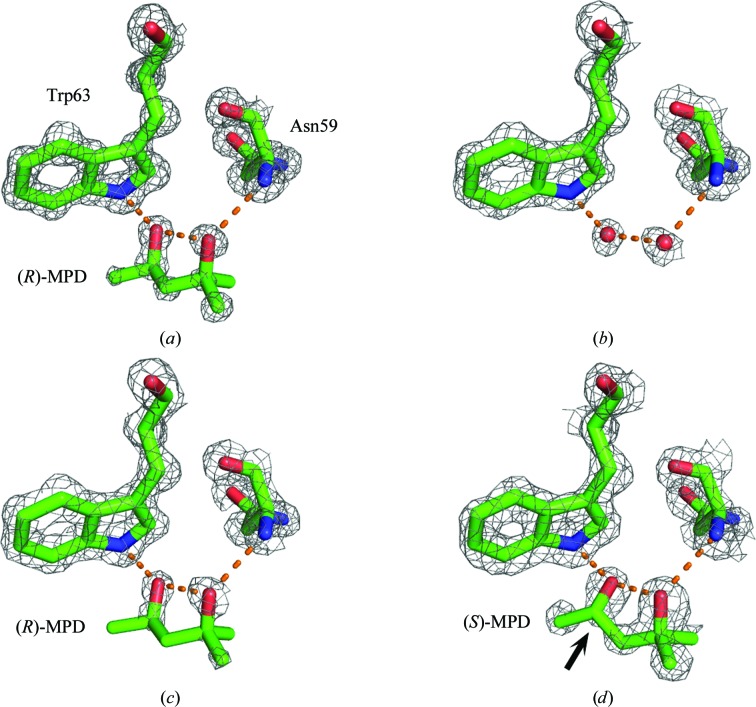
The interaction site near Trp63. (*a*)–(*c*) depict the results from the current work with different additives: (*a*) (*R*)-MPD; (*b*) (*S*)-MPD; (*c*) (*RS*)-MPD. The red spheres in (*b*) are the O atoms of water molecules. For comparison, the results of Michaux *et al.* (2008 ▶), which used (*RS*)-MPD, are also presented (*d*). The 2*F*_o_ − *F*_c_ density is shown as a gray mesh contoured at 1.5σ, except for the (*S*)-MPD molecule in (*d*), which is shown at 1.0σ. This is to highlight that C4 (denoted by an arrow) protrudes from the density.

Here, MPD forms hydrogen bonds to Trp63 and Asn59 of the same protein. Even though it is not a crystal contact site, it may contribute to the differences observed between the various crystals because we find (*R*)-MPD at this site (occupancy 0.60) when it is the additive (Fig. 7 ▶ *a*), but no (*S*)-MPD when it is the sole additive (Fig. 7 ▶ *b*). Instead, we find two water molecules making equivalent hydrogen bonds to those found with (*R*)-MPD. Our (*RS*)-MPD results are consistent with this finding: we observe (*R*)-MPD at the site (Fig. 7 ▶ *c*) with an occupancy of 0.50.

Weiss *et al.* (2000 ▶) find poorly defined electron density in this region, which they assign to water molecules. Michaux *et al.* (2008 ▶) observe more clearly defined density that they fit with (*S*)-MPD (Fig. 7 ▶ *d*), but for this assignment C4 protrudes from the electron density (black arrow in Fig. 7 ▶ *d*). As with the Phe34 site, we believe that (*R*)-MPD is a more appropriate assignment.

It is an open question as to why only (*R*)-MPD is observed at this site. We do not find any obvious factors that would exclude the other enantiomer, as the region near Trp63 does not appear to be a traditional enantioselective binding site for MPD, a pocket in which only one enantiomer fits (Ali *et al.*, 2006 ▶; Haginaka, 2008 ▶). It is likely that more subtle effects are involved in the formation of a preferred diastereomeric complex between the additive and the protein (Lämmerhofer, 2010 ▶).

#### 3.3.3. Trp123 interaction site   

**Figure 8:**
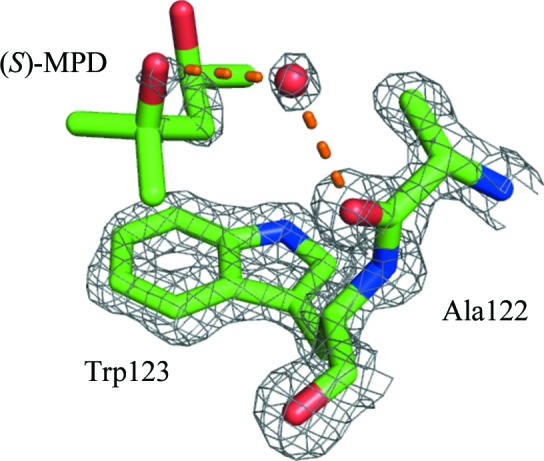
The interaction site near Trp123. (*S*)-MPD interacts with the protein through hydrogen bonds (dashed orange lines). The red sphere is the O atom of a water molecule. The 2*F*_o_ − *F*_c_ density is shown as a gray mesh contoured at 1.5σ. For clarity, the intramolecular hydrogen bond between O2 and O4 of (*S*)-MPD is not shown.

The only crystals in which we can confidently assign MPD at this site are those grown with (*S*)-MPD, where the occupancy is 0.50 (Fig. 8 ▶ and Supplementary Fig. S14). For (*R*)-MPD there is no significant density, while for (*RS*)-MPD the density is too poorly defined to make a definite assignment: we chose to assign water molecules. Weiss *et al.* (2000 ▶) and Michaux *et al.* (2008 ▶) also find poorly defined density at this site; the former group assign a Tris molecule, while the latter assign a water molecule.

It is not clear what role this site plays in the crystal structure. There are no direct hydrogen bonds between MPD and the protein and only one indirect hydrogen bond to Ala122 through a water molecule. Furthemore, as with the Trp63 site, the mechanism of the enantioselective interaction between the protein and MPD remains to be elucidated.

#### 3.3.4. Crystal quality   

The enantioselective interactions of lysozyme with MPD affect the crystal quality. We find the highest resolution and least-disordered crystals (as measured by the average protein *B* factor and the mosaicity) are obtained with (*R*)-MPD (Table 6 ▶). This is true whether we use the maximum resolution-limit data (Table 6 ▶) or compare the crystals at constant *I*/σ(*I*) (Supplementary Table S7). The higher quality of the crystals obtained with (*R*)-MPD is consistent with the better crystal contact that this enantiomer forms with the protein at the Phe34 site.

The other crystals are lower in quality. Those grown with (*S*)-MPD appear to be poorer quality overall, suggesting that (*S*)-MPD has a deleterious effect on crystal growth relative to not using any MPD. Data from a second set of crystals (Supplementary Table S7) support this suggestion.

The crystals grown with (*RS*)-MPD by Weiss *et al.* (2000 ▶) and Michaux *et al.* (2008 ▶) have lower resolution and higher *B* factors than our (*RS*)-MPD crystals, which is probably owing in part to the less pure lysozyme that they used. (These authors do not report the mosaicity in their work, but in any case it would be difficult to compare mosaicities across beamlines as the X-ray beams have different divergences.) It is likely, however, that the use of a racemic additive as opposed to one that is enantiomerically pure also adversely affects the crystal quality. When crystallizing thaumatin with the stereoisomers of sodium tartrate, we found that the highest quality crystals were formed with enantiomerically pure precipitants (Asherie *et al.*, 2009 ▶).

### 3.4. Chirality and protein crystallization: comments and recommendations   

The use of chirality to influence the crystallization of small molecules is an active field of study with a distinguished history (Pérez-García & Amabilino, 2002 ▶). Indeed, Louis Pasteur discovered the molecular basis of chirality in 1848 through a crystallization experiment (Gal, 2008 ▶, 2011 ▶). In contrast, much less work has been performed on the role of chirality in protein crystallization. Apart from our own research, the only other systematic approach that we are aware of that uses chirality to control protein crystallization is the racemic crystallization of synthetic proteins (Pentelute *et al.*, 2008 ▶; Sawaya *et al.*, 2012 ▶; Yeates & Kent, 2012 ▶). If the protein of interest is small enough and has a sufficient number of disulfide bonds to ensure proper folding, it can be produced by total chemical synthesis. Since the protein is assembled artificially, it can be made as two enantiomers, one consisting of naturally occurring l-amino acids and the other with d-amino acids, and then crystallized as enantiomeric pairs. This approach has made it easier to crystallize and solve the structure of more than a dozen proteins (Yeates & Kent, 2012 ▶).

Further indications that chirality is a useful tool in protein crystallization can be gleaned from the literature. For example, to produce high-resolution crystals of the membrane-protein complex photosystem I in β-dodecylmaltoside, the lipid must be of sufficient stereochemical purity: the α-stereoisomer content must be below 10% (Fromme & Witt, 1998 ▶). One difficulty that arises during a literature search is that the stereochemical identity of the additives used is often omitted by the manufacturer (especially in the case of crystallization kits) or by the investigator. We note that chiral molecules are commonly found in commercially available kits. In the 180 kits we examined from eight manufacturers (Hampton Research, Qiagen, Jena Biosciences, Molecular Dimensions, Sigma–Aldrich, Microlytic, Anatrace and Rigaku), we found at least one chiral molecule in 134 kits (74% of all kits). Given the widespread inclusion of chiral molecules in kits and the possible usefulness of chirality in a protein crystallization experiment, we encourage other investigators to specify the absolute stereochemical configuration of all chemicals used when reporting their experimental results.

In addition to a general lack of information about chirality, we also encountered nomenclature problems related to how the molecule 2-methyl-2,4-pentanediol is described in the PDB. Inconsistent numbering schemes are often used, *e.g.* in PDB entry 1jlt, where the two MPD molecules in the structure are numbered differently. In particular, CM and C1 are regularly switched, making it harder to analyze the conformations of the additives and obscuring the structural information that one molecule is the mirror image of the other. The numbering we chose (Fig. 1 ▶) follows the IUPAC recommendation for branched hydrocarbons (IUPAC, 1979 ▶) and appropriately highlights the **1a** conformation. In fact, we had to repeatedly request the PDB to use this numbering for (*R*)-MPD in our structures, as when we initially deposited them the numbering was switched. The tendency of the PDB to perpetuate incorrect or confusing numbering schemes has been recently noted (Jaskolski, 2013 ▶) and should be corrected.

Another problem with nomenclature and chiral molecules in the PDB arises with the three-letter ligand IDs used to identify the molecules. At the time of writing, a search of the PDB for ‘MPD’, which is the ligand ID for (*S*)-MPD, finds 826 structures; a search for ‘MRD’, the ligand ID for (*R*)-MPD, finds only 309 structures. This difference may be owing to a real chiral effect, but since most of the structures are not high resolution, it is unlikely that this effect can always be seen. That is, the density for the ligand could be fitted with either enantiomer. [For all experiments apart from ours, (*RS*)-MPD is the additive used, so in principle either enantiomer could be present in the X-ray structure.] Indeed, for the high-resolution data we analyze we find almost equal numbers of (*R*)- and (*S*)-MPD molecules interacting with proteins. The preponderance of (*S*)-MPD in the PDB probably reflects a linguistic bias: when people fit their data, they use MPD, *i.e.* (*S*)-MPD, because it is the acronym by which most people refer to the molecule 2-methyl-2,4-pentanediol. To avoid such problems, we suggest that the PDB should name enantiomers, and more generally stereoisomers, using abbreviations that do not introduce bias. This is particularly important given the large number of chiral molecules in the PDB.

There are 90 chiral molecules in the top 200 PDB ligands (ranked by ligand hits, *i.e.* the number of times the ligand is reported in a PDB structure) and approximately 20% of ligand hits involve chiral molecules. We believe that since chiral molecules are common, possible chiral effects in protein crystallization should be explored in detail. Furthermore, it seems reasonable to explore more general stereochemical effects beyond enantiomerism. Given the prevalence of sugars as ligands in the PDB, we consider the stereoisomerism of sugars as an interesting possibility to consider when crystallizing proteins.

While the PDB protein structures and ligand list offer a useful starting point for choosing candidate chiral ligands, they provide only a partial view of the role of chiral molecules in protein crystallization. It is possible for chiral effects to be present in solution during protein nucleation but that the final crystal does not incorporate the chiral additive. Indeed, we have observed this with thaumatin and tartrate. The addition of l-tartrate to thaumatin produces bipyramidal crystals that incorporate the additive into the lattice. The crystals have normal solubility and a tetragonal space group. Addition of d-tartrate leads instead to the formation of prismatic crystals with retrograde solubility and an orthorhombic space group; these crystals do not contain any tartrate (Asherie, Ginsberg, Blass *et al.*, 2008 ▶; Asherie, Ginsberg, Greenbaum *et al.*, 2008 ▶; Asherie *et al.*, 2009 ▶).

We focus here on high-resolution structures (resolution of better than 1.5 Å) because they allow us to determine the stereochemistry and conformation of MPD with minimal uncertainty and therefore we are able to analyze chiral effects in detail. By doing so, we do not mean to imply that chiral effects are confined only to high-resolution structures. On the contrary, chiral effects span the range from the obvious (some of which can be seen with the naked eye) to the subtle. This is well known in small-molecule systems and by studying different protein–additive pairs we expect to find that it holds for protein systems as well.

Further work is needed to fully understand the mechanism by which chirality affects protein crystallization. Chiral effects are often clear at crystal contacts, but these only account for only a small part of protein–additive interactions: we estimate the fraction of protein structures with at least one crystal contact by a chiral molecule to be about 5% (Carugo & Djinović-Carugo, 2014 ▶). A more common situation is one in which two enantiomers interact with the protein at different sites, but these are not crystal contact sites; this is the case for the Trp63 and Trp123 interaction sites discussed in this work. Also, as we mentioned above, chiral molecules can have an effect in the solution phase.

We appreciate that working with enantiomerically pure additives is expensive. Cost is one possible reason why crystallization experiments with MPD have thus far been carried out only with the racemate. At the time of writing, the cost per gram of 99% pure (*R*)-MPD from Sigma–Aldrich (catalog No. 252840) is more than 3000 times that of (*RS*)-MPD of similar purity (catalog No. 112100). Nevertheless, given the potential benefits, some way to assess chiral effects should be incorporated into a crystallization experiment, and we expect that cost will diminish with increased demand for enantiomerically pure additives. If a full screening of crystallization conditions with the separate enantiomers of the additive under study is prohibitively expensive, we suggest that the initial screen be carried out with the cheaper racemate; promising conditions may then be optimized with the pure enantiomers. We are happy to provide small amounts of pure (*R*)- and (*S*)-MPD to members of the community.

## 4. Conclusions   

We crystallized lysozyme with (*R*)-, (*S*)- and (*RS*)-MPD. We also grew crystals without MPD under similar conditions. All four crystalline arrangements obtained have the same space group and almost identical unit-cell parameters. The crystals grown with (*R*)-MPD have the highest resolution and least disorder, suggesting a preferential interaction between lysozyme and this enantiomer of MPD. This idea is confirmed by the X-ray structures, which show that the two enantiomers interact differently with the protein. Our findings support the hypothesis that chiral interactions with chiral additives are important in protein crystallization.

## 5. Related literature   

The following references are cited in the Supporting Information for this article: Ewing *et al.* (1996 ▶) and Lomakin *et al.* (2005 ▶).

## Supplementary Material

PDB reference: lysozyme, crystallized with (*RS*)-2-methyl-2,4-pentanediol, 4b4j

PDB reference: crystallized with (*R*)-2-methyl-2,4-pentanediol, 4b4e

PDB reference: crystallized with (*S*)-2-methyl-2,4-pentanediol, 4b4i

PDB reference: crystallized without 2-methyl-2,4-pentane­diol, 4b49

Supplementary Material. DOI: 10.1107/S1399004714025061/nj5208sup1.pdf

## Figures and Tables

**Table 1:** Characterization of (*R*)-, (*S*)- and (*RS*)-MPD

	Chemical purity (%)	Enantiomeric ratio	_D_^20^ (*c* 1.0, H_2_O)
(*R*)-MPD	99.5	99.9:0.1	18.8 0.2
(*S*)-MPD	99.7	99.9:0.1	19.0 0.2
(*RS*)-MPD	99.9	50:50†	0.0 0.2

† Theoretical value (not measured).

**Table 2:** Relative energies of the conformers of (*R*)-MPD from QC calculations

Conformer	*E* (kJmol^1^) *in vacuo*	*E* (kJmol^1^) in water (PCM†)
**1a **	0.00	0.00
**3a**	12.41	14.93
**2a **	13.86	15.17
**3c**	14.46	15.91
**3b **	17.40	17.46
**1b **	19.53	19.40
**2b **	23.19	20.74
**1c**	23.45	24.48
**2c**	27.77	27.40

† PCM: polarizable continuum model.

**Table 3:** Relative Helmholtz free energies of the conformers of (*R*)-MPD from MD simulations

Conformer	*F* (kJmol^1^), 300K	*F* (kJ mol^1^), 370K
**1a **	0.00	0.00
**2a **	5.93	3.41
**3b **	7.55	5.89
**3a **	7.92	6.46
**2b **	7.99	5.73
**1b **	8.75	8.16
**1c **	20.07	18.88
**2c **	†	17.54
**3c**	†	18.45

† Conformer not observed.

**Table 4:** Relative Gibbs free energies of the conformers of (*R*)- and (*S*)-MPD extracted from the PDB

Conformer	*G* (kJmol^1^), 300K
**1a **	0.00
**2a **	3.70
**3a **	4.71
**3c**	6.99
**1b **	7.33
**1c **	7.33
**2c **	7.33
**2b**	7.71
**3b**	8.72

**Table 5:** Crystallographic data and refinement statistics

PDB entry	4b49	4b4e	4b4i	4b4j
MPD added	None	(*R*)	(*S*)	(*RS*)
Data collection				
Space group	*P*4_3_2_1_2	*P*4_3_2_1_2	*P*4_3_2_1_2	*P*4_3_2_1_2
Unit-cell parameters				
*a* = *b* ()	76.84	77.53	77.44	77.67
*c* ()	38.69	37.89	37.94	37.70
= = ()	90	90	90	90
Resolution† ()	20.001.15 (1.171.15)	15.001.00 (1.021.00)	30.001.20 (1.221.20)	30.001.25 (1.271.25)
Total reflections	562743	853125	512028	456741
Unique reflections†	41718 (2040)	62397 (3076)	36597 (1806)	32449 (1584)
*R*_merge_ †	0.08 (0.76)	0.07 (0.82)	0.08 (0.79)	0.06 (0.56)
*I*/(*I*)†	41.5 (3.2)	43.3 (2.5)	45.1 (3.6)	45.9 (5.5)
Completeness† (%)	99.8 (99.1)	99.3 (99.0)	99.7 (100.0)	99.8 (99.6)
Multiplicity†	13.5 (10.9)	13.7 (11.2)	14.0 (12.7)	14.1 (13.2)
Mosaicity ()	0.32	0.26	0.46	0.43
Overall *B* factor (^2^)	15.5	13.4	17.2	15.1
*V*_M_ (^3^Da^1^)	2.03	2.03	2.03	2.03
Solvent content (%)	39.6	39.6	39.4	39.3
Refinement				
Resolution† ()	18.671.15 (1.181.15)	13.301.00 (1.031.00)	21.491.20 (1.231.20)	27.051.25 (1.281.25)
Reflections [*R*_cryst_ + *R*_free_ (5%)]	39425 + 2089	59136 + 3155	34682 + 1825	30732 + 1643
*R*_cryst_/*R*_free_ † (%)	12.7/15.1 (22.2/25.6)	12.4/14.4 (25.0/25.7)	12.9/16.7 (18.8/22.1)	13.05/15.57 (18.1/22.7)
No. of atoms				
Protein (No. of residues)‡	1001 (129)	1000 (129)	1000 (129)	1001 (129)
MPD§		16 (2; *R*)	16 (2; *S*)	16 (2; *R*)
Ions/ligands (No. of molecules)	11 (4)	3 (3)	2 (2)	2 (2)
Water	267	196	188	162
*B* factors (^2^)				
Protein	12.9	11.9	15.2	13.7
MPD		12.3	21.4	13.1
Ions/ligands	13.1	14.2	17.7	15.4
Water	26.2	21.6	28.3	24.6
R.m.s. deviation from ideal				
Bond lengths ()	0.009	0.008	0.007	0.007
Bond angles ()	1.349	1.306	1.214	1.272
Ramachandran plot				
Most favored (%)	90.3	90.3	88.5	87.6
Additionally favored (%)	9.7	9.7	11.5	12.4
Disallowed (%)	0.0	0.0	0.0	0.0

† Values in parentheses are for the highest resolution shell.

‡ Atom OXT (residue Leu129) was omitted during the refinement for structures 4b4e and 4b4j.

§ The number of molecules in the structure and the enantiomer are given in parentheses.

**Table 6:** Comparison of crystal quality

Investigators	MPD additive	Maximum resolution ()	Overall *B* factor (^2^)	Mosaicity ()
This work	(*R*)-MPD	1.00	13.4	0.26
This work	None	1.15	15.5	0.32
This work	(*S*)-MPD	1.20	17.2	0.46
This work	(*RS*)-MPD	1.25	15.1	0.43
Weiss and coworkers†	(*RS*)-MPD	1.64	19.0	
Michaux and coworkers‡	(*RS*)-MPD	1.75	20.2	

† PDB entry 1dpw (Weiss *et al.*, 2000 ▶).

‡ PDB entry 3b72 (Michaux *et al.*, 2008 ▶).
