# Supplementary material for: Crystallization of lysozyme with (R)-, (S)- and (RS)-2-methyl-2,4-pentanediol
Source: Acta Crystallogr D Biol Crystallogr. 2015 Feb 26;71(Pt 3):427–41. doi: 10.1107/S1399004714025061 (PMC4356360; doi:10.1107/S1399004714025061)
Supplement: Supplementary file 1 [file d-71-00427-sup1.pdf]

BIOLOGICAL CRYSTALLOGRAPHY

Volume 70 (2014)

Supporting information for article:

Crystallization of lysozyme with (*R*)-, (*S*)- and (*RS*)-2-methyl-2,4-pentanediol

Mark Stauber, Jean Jakoncic, Jacob Berger, Jerome M. Karp, Ariel Axelbaum, Dahniel Sastow, Sergey V. Buldyrev, Bruce J. Hrnjez and Neer Asherie

S1. Protein characterization

S1.1. Size-exclusion and cation-exchange chromatography

Both size-exclusion and cation-exchange high-performance liquid chromatography (SE-HPLC and CE-HPLC, respectively) were carried out on a Beckman System Gold apparatus at a flow rate of 1 ml/min (Beckman Coulter, Fullerton, CA). SE-HPLC measurements were carried out by isocratic elution on a Superdex 75 10/300 GL column (GE Healthcare, Piscataway, NJ). The buffer used was 100 mM sodium phosphate buffer (pH 7.1, σ = 11.4 mS/cm) with 0.02% (m/v) sodium azide. CE-HPLC measurements were carried out on a Bakerbond Wide-Pore (5 μm particle size) CBx column (Mallinckrodt Baker, Phillipsburg, NJ). The proteins were eluted in 20 mM Tris acetate containing 0.02% (m/v) sodium azide (pH 6.5, σ = 1.5 mS/cm) with a salt gradient of 0-100% 0.5 M sodium acetate (pH 6.5, σ = 30.5 mS/cm) for 41 minutes.

S1.2. Quasielastic light scattering

Quasielastic light scattering (QLS) was performed on a home-built optical system using a 35 mW He-Ne (633nm) laser (Coherent, Santa Clara, CA), a custom-made scattering cell (Precision Detectors, Bellingham, MA), and a PD2000DLS^{PLUS} 256 channel correlator (Precision Detectors, Bellingham, MA). The scattering angle was 90° and all measurements were carried out at 20 ± 0.3 °C. The measured correlation functions were analyzed by a constrained regularization method as implemented in the PrecisionDeconvolve software (version 5.4) provided by Precision Detectors. This software computes the distribution of scattered intensity as a function of the diffusion coefficient. To convert the diffusion coefficient to a hydrodynamic radius (Lomakin *et al.*, 2005), the viscosity of the solution was taken to be that of water (1.002 mPa s). The QLS measurements were done in 275 mM acetate buffer (pH = 4.5, σ = 8.0 mS/cm) with 0.02% (m/v) sodium azide, and all samples were filtered through an Anotop 10 0.02 μm filter (Whatman, Maidstone, UK).

S1.3. Electrospray ionization mass spectroscopy

Samples were dialyzed into deionized water using Ultrafree-MC centrifugal concentrators (5 kDa membrane; Millipore, Billerica, MA) and then filtered through an Ultra-MC centrifugal filter (0.22 μm; Millipore, Billerica, MA). Mass spectrometry was performed at the Laboratory for

Macromolecular Analysis and Proteomics at the Albert Einstein College of Medicine of Yeshiva University.

S2. MPD characterization

S2.1. NMR

Samples were dissolved in $CDCl_3$ (approximately 40 mg of MPD in about 0.5 ml of solvent). ^{1}H and ^{13}C NMR measurements were performed on a Bruker DRX 300 spectrometer (300 MHz) at the Structural NMR Resource at the Albert Einstein College of Medicine of Yeshiva University.

S2.2. Tandem mass spectrometry (MS/MS)

Samples were dissolved in deionized water (the dilution factor for neat samples was 10^4). MS/MS was performed at the Laboratory for Macromolecular Analysis and Proteomics at the Albert Einstein College of Medicine of Yeshiva University.

S2.3. Specific rotation

Samples were dissolved in deionized water to 10 mg/ml. Specific rotation measurements were performed by Spectrix Analytical Services (North Haven, CT).

S2.4. Gas chromatography (GC)

GC was carried out for (*R*)- and (*S*)-MPD by the manufacturer, Reuter Chemische Apparatebau KG (Freiburg, Germany), on a Shimadzu GC-14 apparatus with a flame ionization detector. Both chemical and enantiomeric purity measurements were performed on an FS-Cyclodex β-I/P column using hydrogen as the mobile phase.

S3. Statistical analysis of molecular dynamics simulations

The (*R*)- and (*S*)-MPD simulation results at a given temperature (300 K and 370 K) were compared to check that they are consistent with the expected mirror symmetries. For each enantiomer, the time series data of the (ψ_1,ψ_2) conformations (recorded every 1 ps during the 100 ns simulations; the first 10 ns were taken to be equilibration time and were not included in the analysis) was used to compute the transition matrix between any two of the bins delimiting the nine conformers. (The bins are

shown in Fig. 3a of the main text.) Using this transition matrix, 1000 realizations of the time series of 90,000 points were simulated and then used to calculate the average probability p_i of observing conformer *i* and its standard deviation σ_i. A chi-square test was used to compare the (*R*)- and (*S*)-MPD results using the following statistic:

$$\chi^2 = \sum_i \frac{\left[p_i(R) - p_i(S)\right]^2}{\sigma_i^2(R) + \sigma_i^2(S)} \quad \text{(S1)}$$

The number of degrees of freedom *m* is given by $m = n - b - 1$, where *n* is the total number of bins and *b* is the number of bins that are empty for both (*R*)- and (*S*)-MPD (Press *et al.*, 1992). Thus, $m = 8$ at 370 K and $m = 6$ at 300 K.

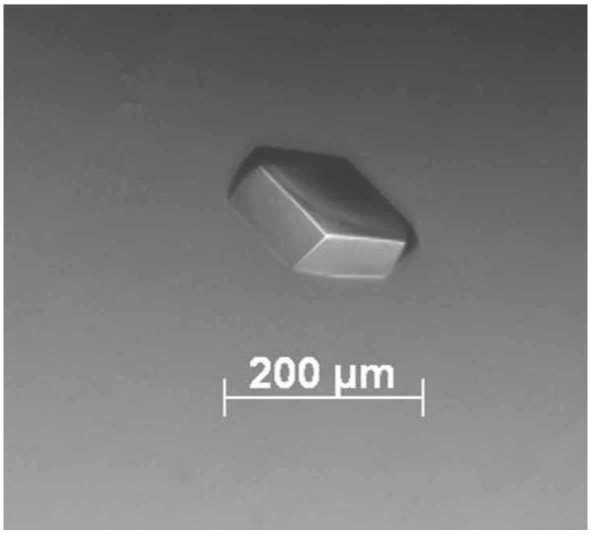

Figure S1. Lysozyme crystal grown with 30% (v/v) (*R*)-MPD at 4°C after 5 days.

Table S1. Properties of atoms used in the molecular dynamics simulations

Atom	OPLS-AA type	Atom type	charge	σ (nm)*	ε (kJ/mol)*
C1, CM, C5	opls_135	alkane **C**H_3	-0.180	0.350	0.276144
H11, H12, H13, HM1, HM2, HM3, H31, H32, H4, H51, H52, H53	opls_140	alkane **H**	0.060	0.250	0.125520
C2	opls_159	alcohol **C**R_2OH	0.265	0.350	0.276144
O2, O4	opls_154	mono alcohol -**O**H	-0.683	0.312	0.711280
HO2, HO4	opls_155	mono alcohol -**O**H	0.418	0	0
C3	opls_136	alkane **C**H_2	-0.120	0.350	0.276144
C4	opls_158	alcohol **C**HROH	0.205	0.350	0.276144

*Parameters of the Lennard-Jones potential.

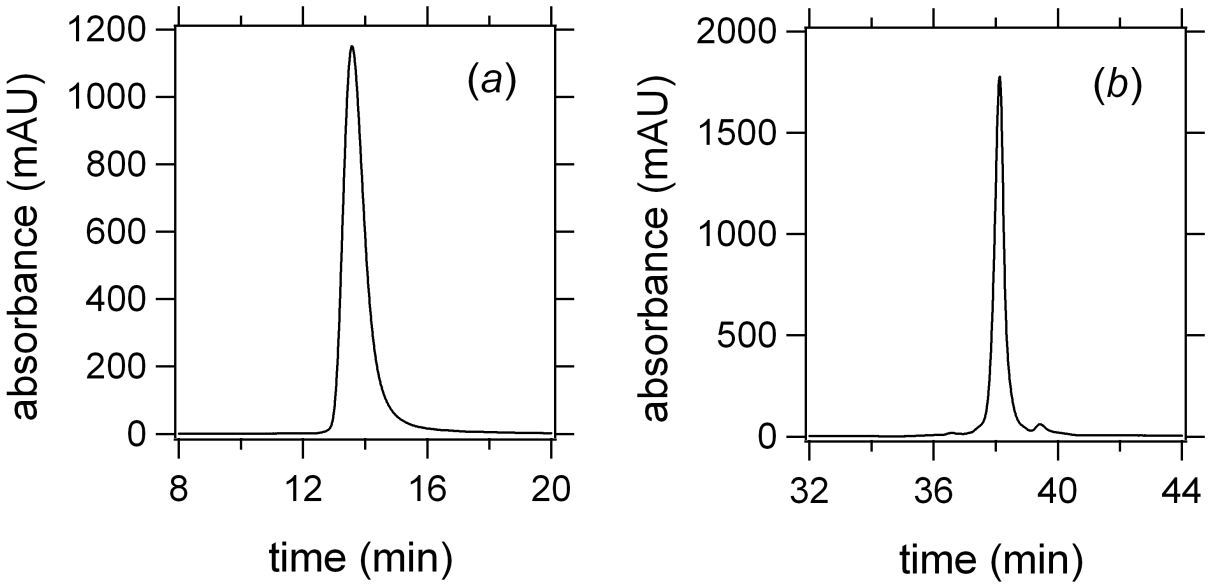

Figure S2. HPLC results for Worthington lysozyme. (*a*) Size-exclusion HPLC chromatogram of lysozyme at 21.7 mg/ml. The protein is essentially monomeric; the slight tailing is due to lysozyme binding to the column (Ewing *et al.*, 1996). (*b*) Cation-exchange HPLC chromatogram for lysozyme at 12.4 mg/ml. The protein is predominantly (> 93%) a single-charge species (major peak at 38.6 min).

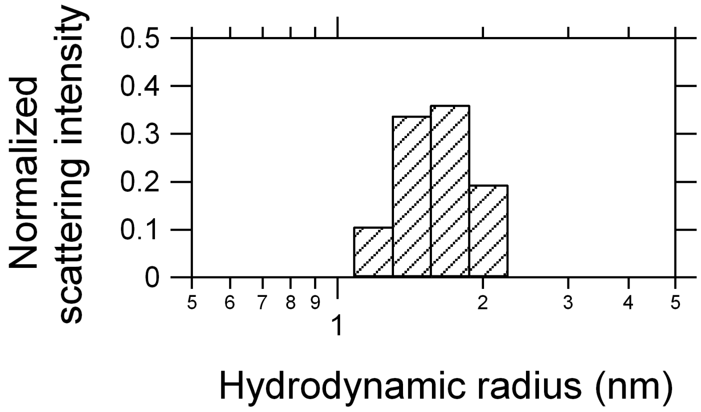

Figure S3. QLS results for lysozyme. The average hydrodynamic radius is 1.77 ± 0.04 nm, which agrees with the value of 1.8 ± 0.2 nm found by other investigators (Parmar *et al.*, 2007).

Table S2. Relative molecular mass of lysozyme

	Found	Calculated*
M_r	14,306.0 ± 1.4	14,305.1

*Obtained from the sequence assuming that all four disulfide bonds of the protein are intact.

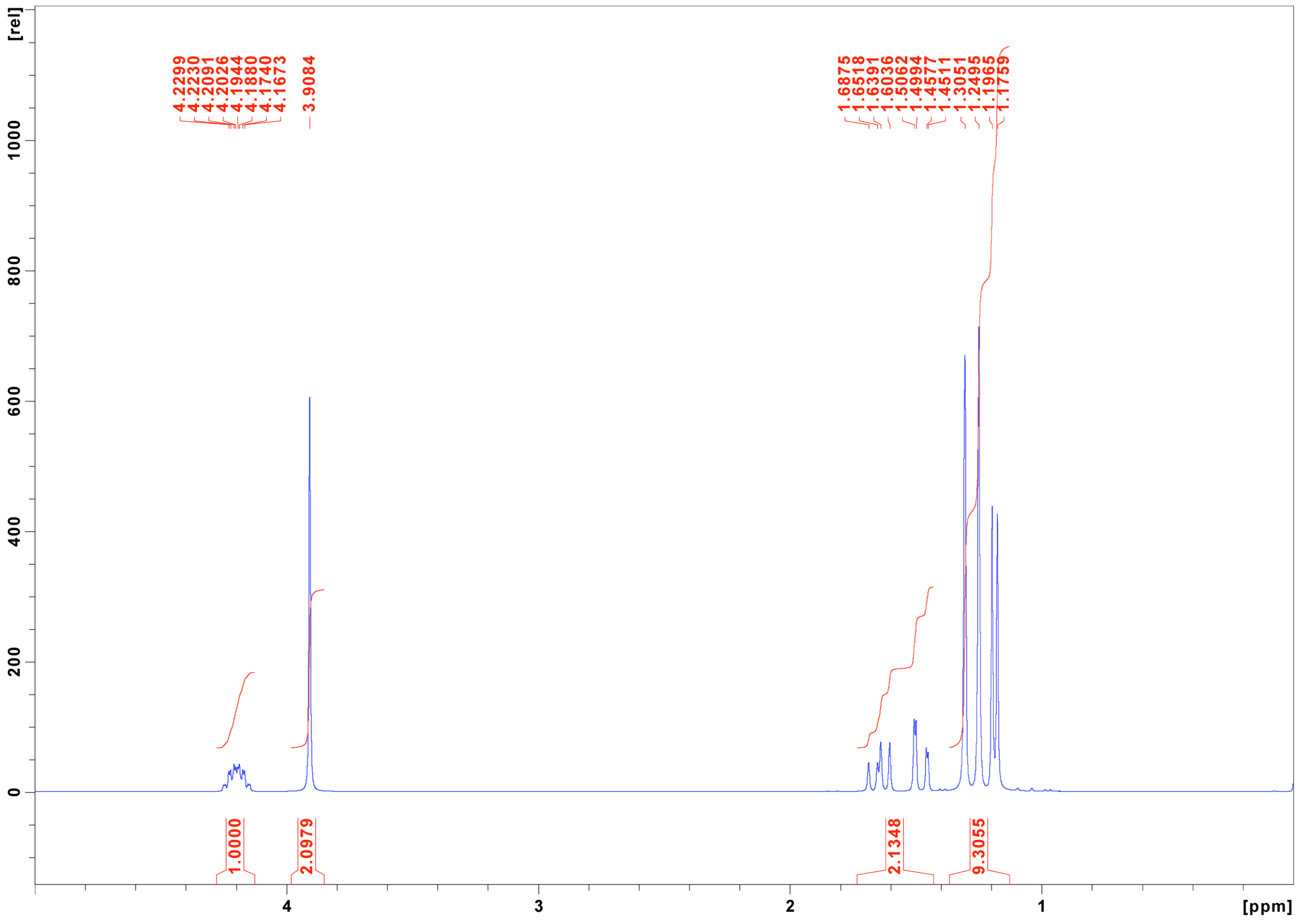

Figure S4. ^{1}H NMR (300 MHz, $CDCl_3$) spectrum for (*R*)-MPD.

(*R*)-MPD resonances: δ 4.23–4.17 (m, 1H), 3.91 (s, 2H), 1.65 (dd, J = 14.5, 10.7 Hz, 1H), 1.48 (dd, J = 14.5, 2.0 Hz, 1H), 1.31 (s, 3H), 1.25 (s, 3H), 1.19 (d, J = 6.2 Hz, 3H).

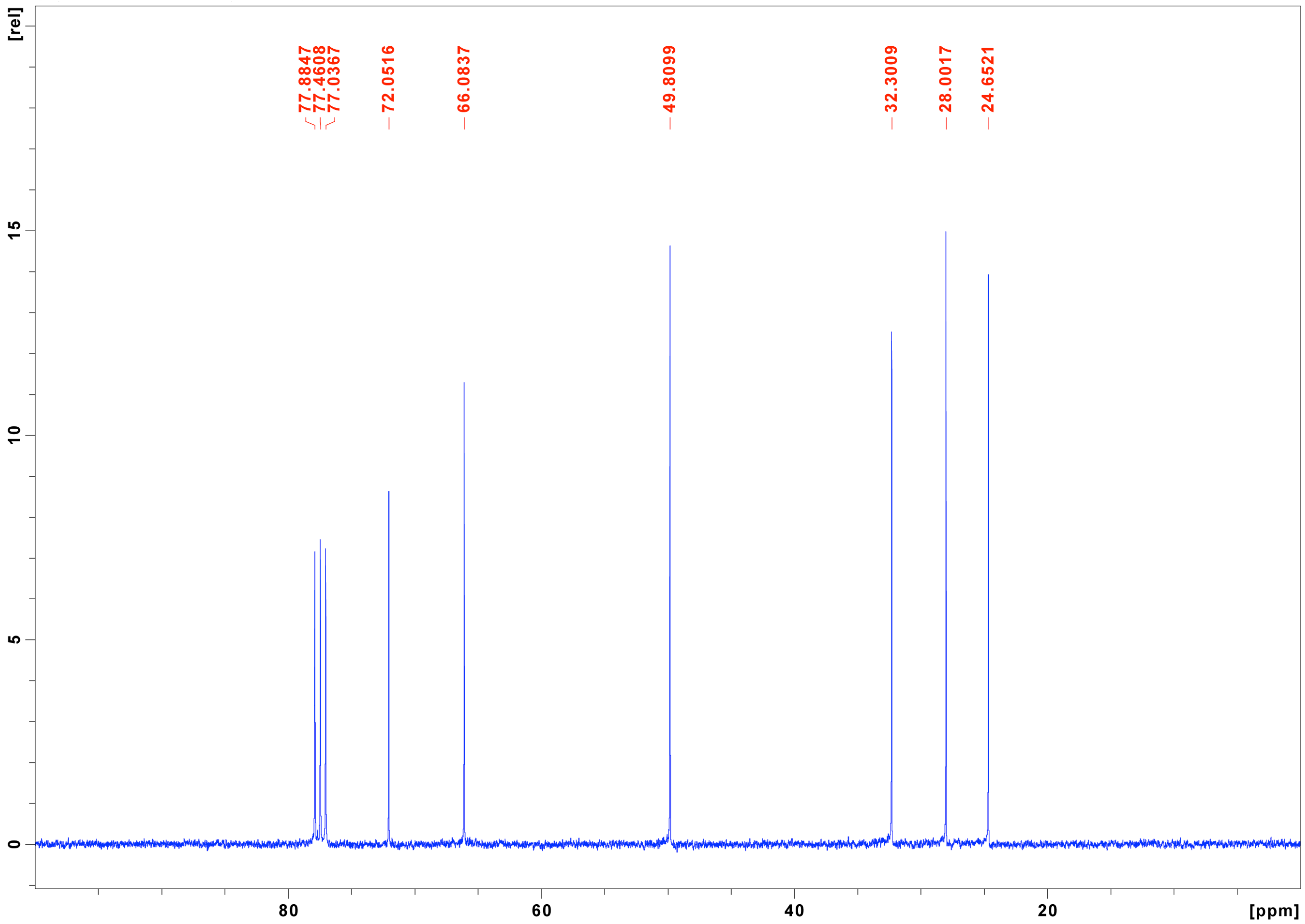

Figure S5. ^{13}C NMR (300 MHz, $CDCl_3$) spectrum for (*R*)-MPD.

(*R*)-MPD resonances: δ 72.05, 66.08, 49.81, 32.30, 28.00, 24.65.

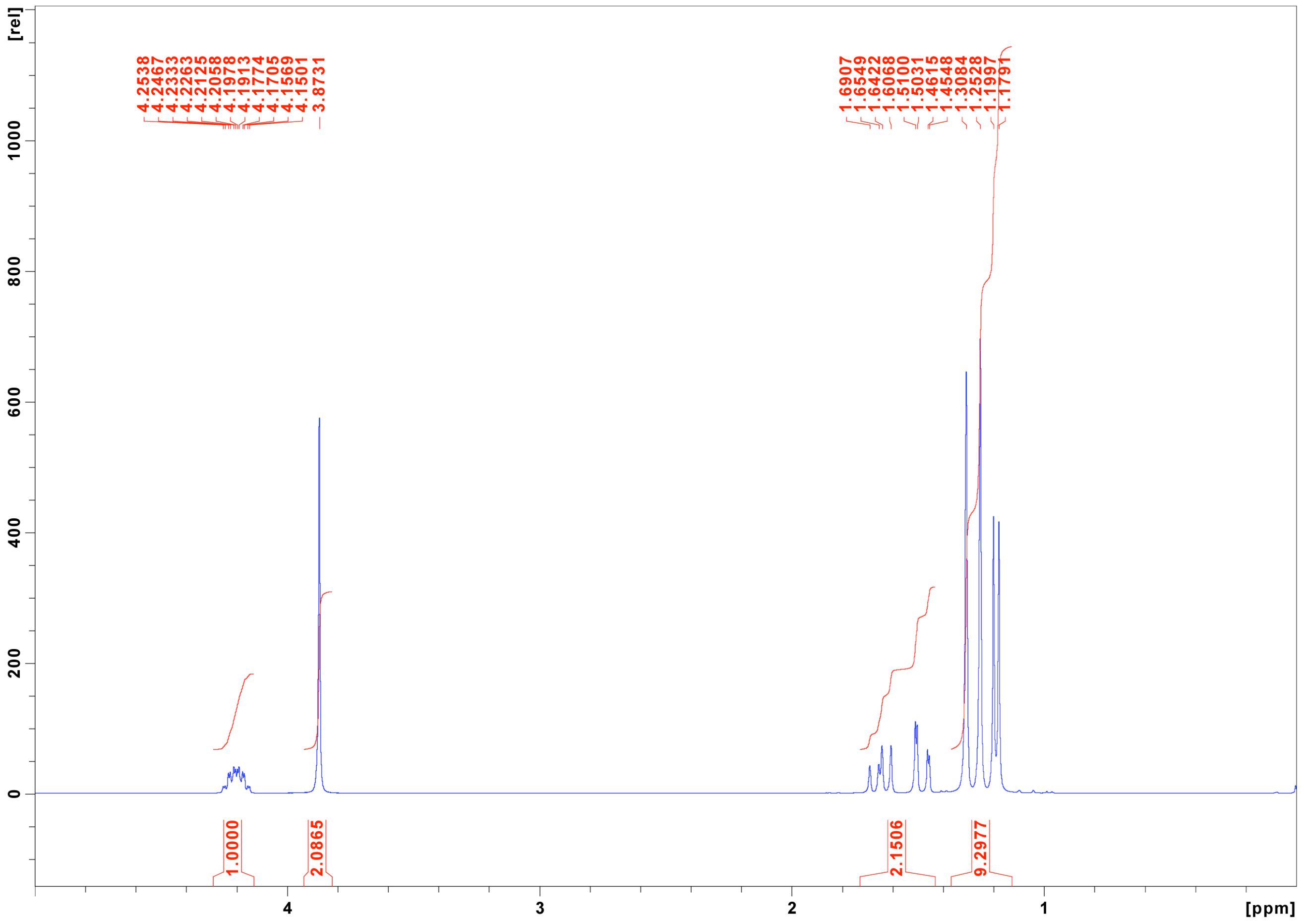

Figure S6. ^{1}H NMR spectrum for (*S*)-MPD.

(*S*)-MPD resonances: δ 4.25–4.15 (m, 1H), 3.87 (s, 2H), 1.65 (dd, J = 14.5, 10.7 Hz, 1H), 1.48 (dd, J = 14.5, 2.0 Hz, 1H), 1.31 (s, 3H), 1.25 (s, 3H), 1.19 (d, J = 6.2 Hz, 3H).

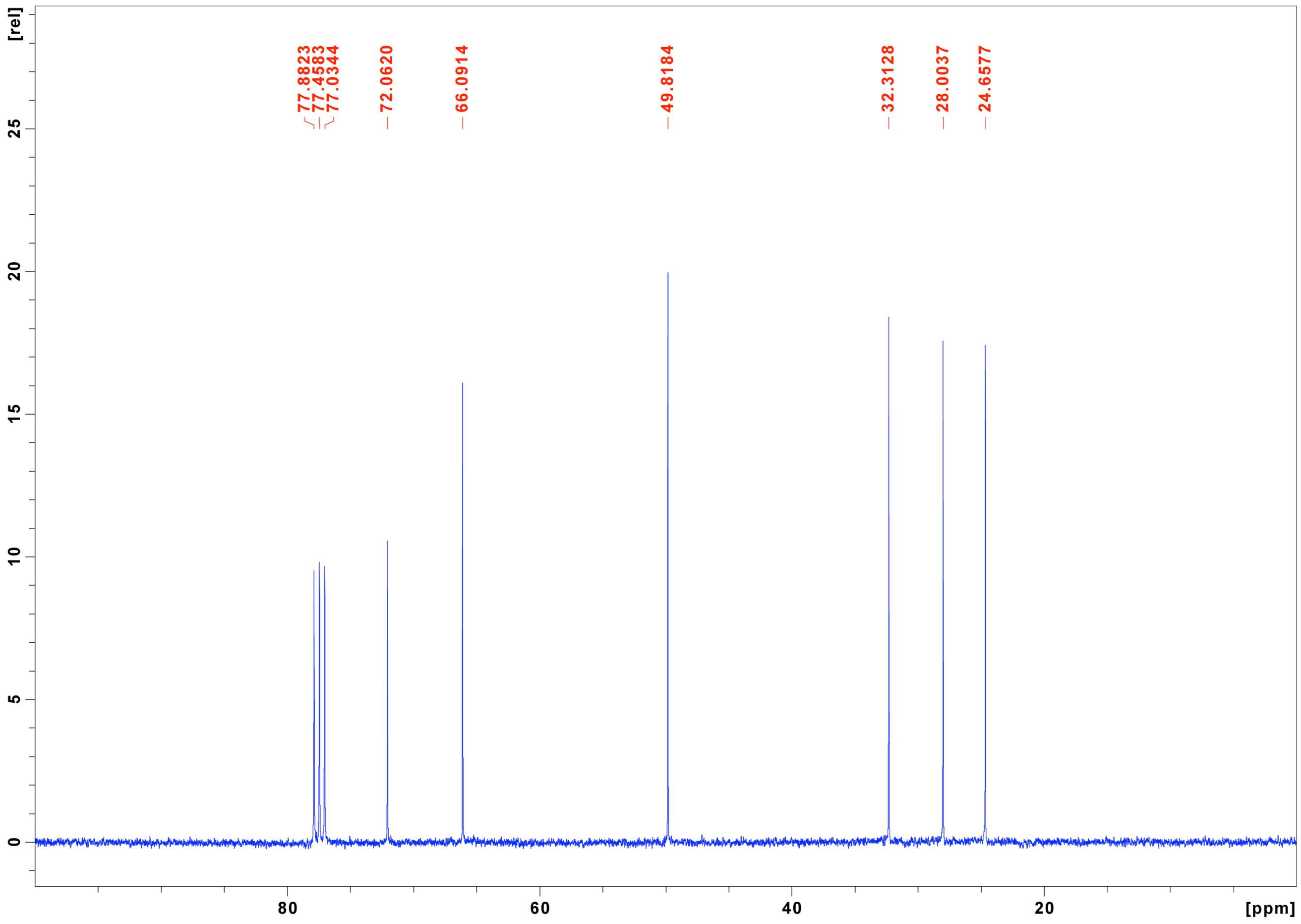

Figure S7. ^{13}C NMR (300 MHz, $CDCl_3$) spectrum for (*S*)-MPD.

(*S*)-MPD resonances: δ 72.06, 66.09, 49.82, 32.31, 28.00, 24.66.

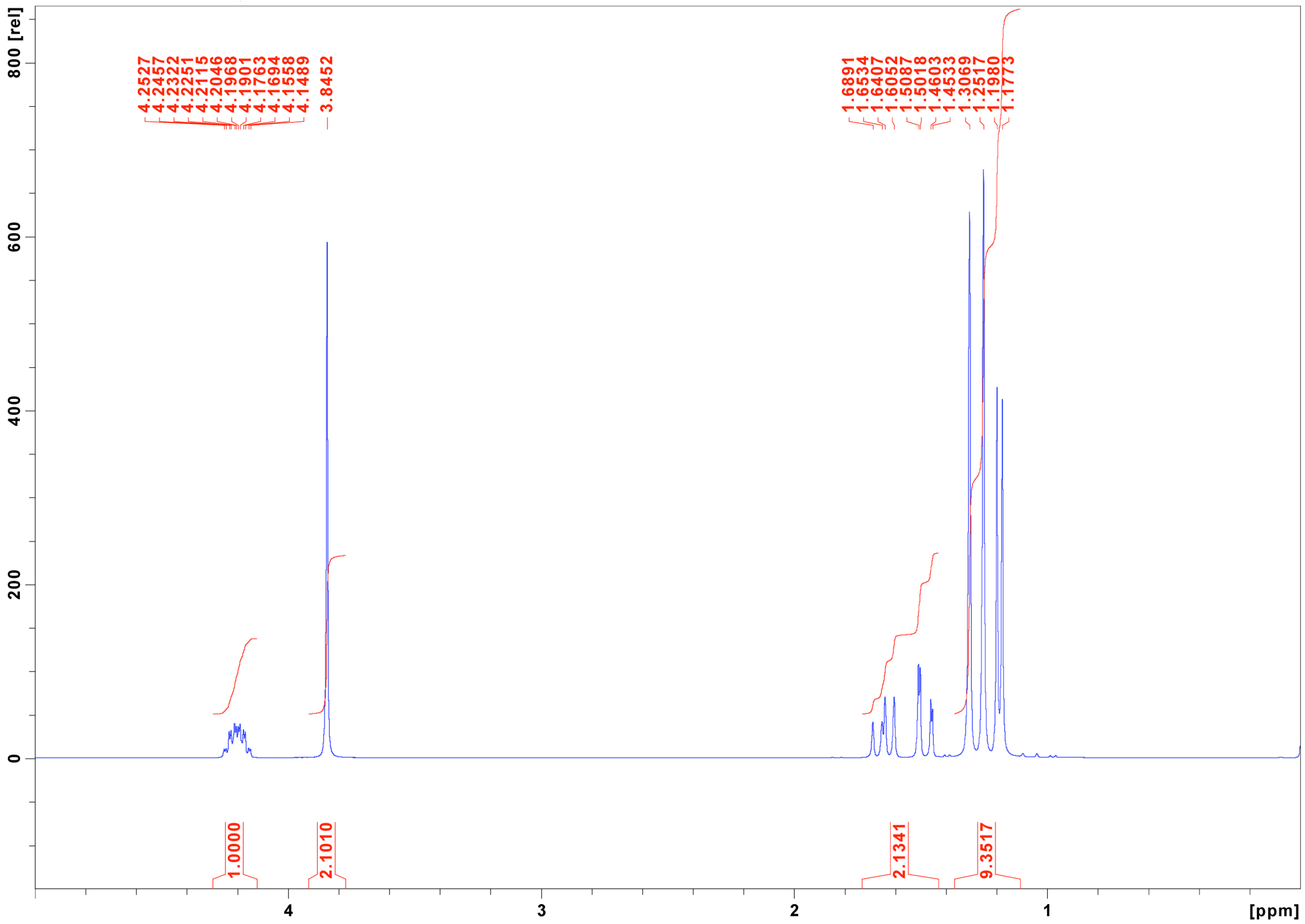

Figure S8. ^{1}H NMR spectrum for (*RS*)-MPD.

(*RS*)-MPD resonances: δ 4.25–4.15 (m, 1H), 3.85 (s, 2H), 1.65 (dd, J = 14.5, 10.7 Hz, 1H), 1.48 (dd, J = 14.5, 2.1 Hz, 1H), 1.31 (s, 3H), 1.25 (s, 3H), 1.19 (d, J = 6.2 Hz, 3H).

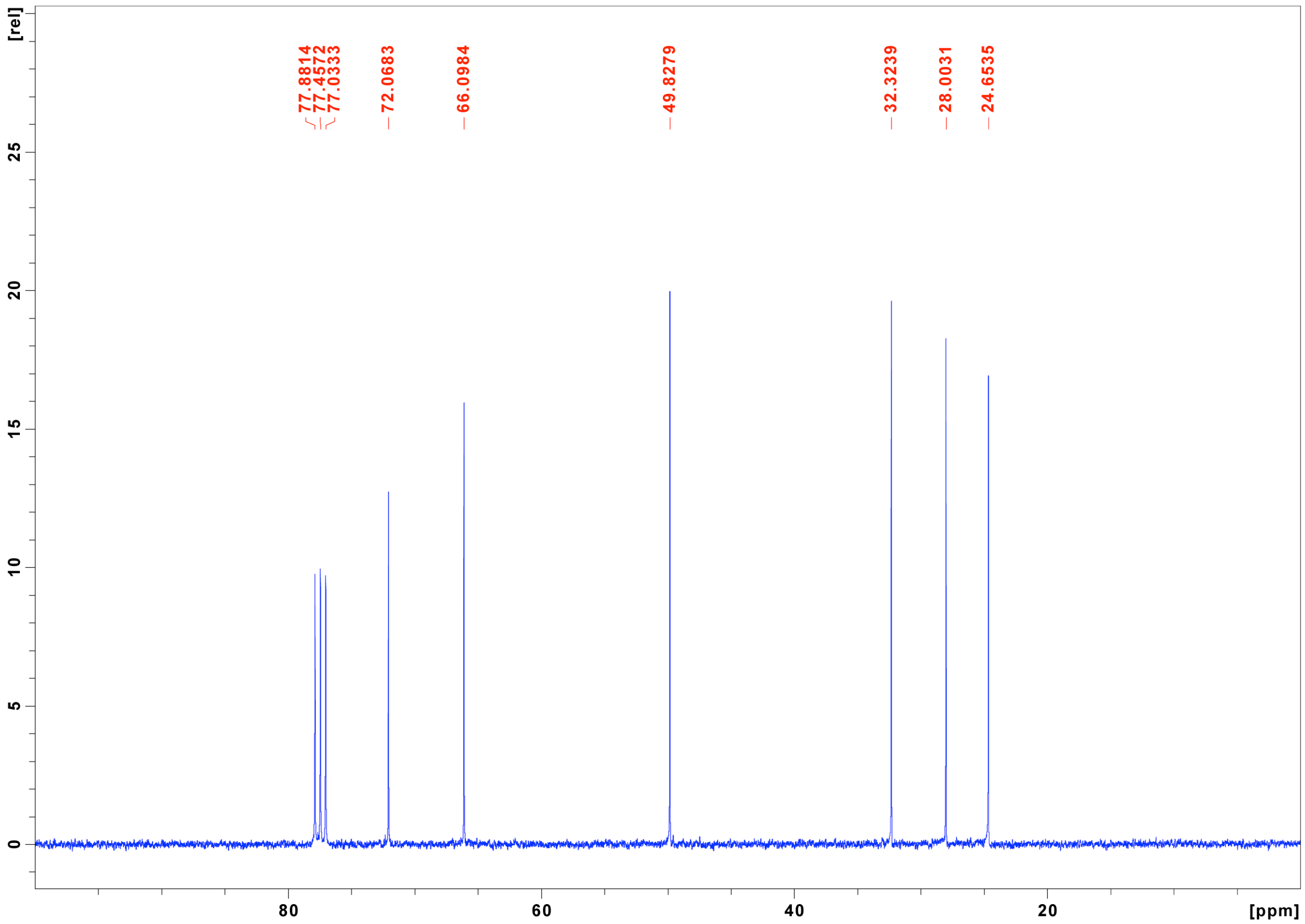

Figure S9. ^{13}C NMR (300 MHz, $CDCl_3$) spectrum for (*RS*)-MPD.

(*RS*)-MPD resonances: δ 72.07, 66.10, 49.83, 32.32, 28.00, 24.65.

Table S3. MS/MS results for MPD

Values of *m/z* for the monoisotopic singly charged species MH^+

	Found	Calculated
(*R*)-MPD	119.1091	119.1067
(*S*)-MPD	119.1085	119.1067
(*RS*)-MPD	119.1098	119.1067

Table S4. Specific rotation of MPD

	$[\alpha]_D^{20}$ (c 1.0, H_2O)
(*R*)-MPD	-18.8 ± 0.2
(*S*)-MPD	19.0 ± 0.2
(*RS*)-MPD	0.0 ± 0.2

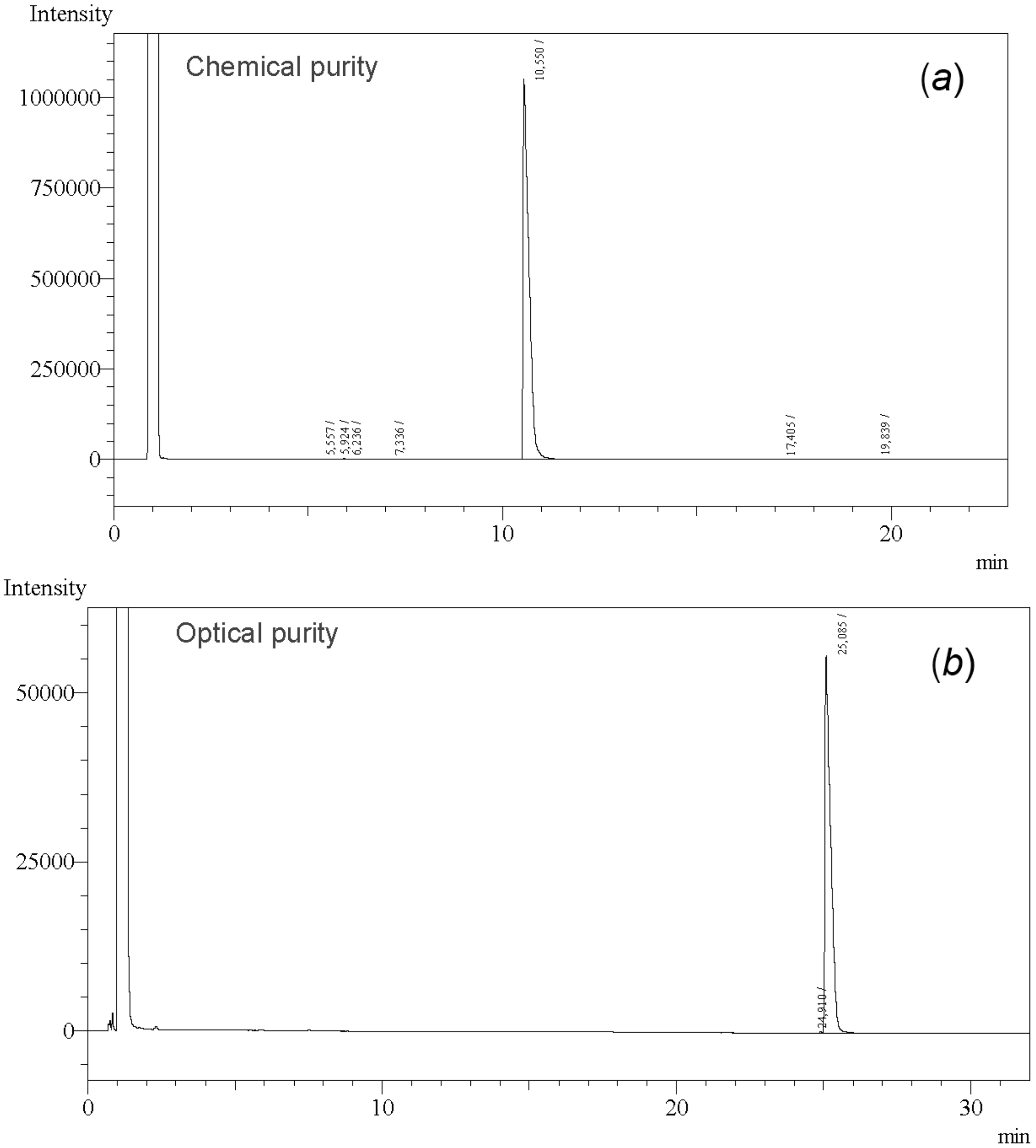

Figure S10. GC measurements of (*a*) chemical and (*b*) optical purity for (*R*)-MPD. The chemical purity is 99.5%; the enantiomeric ratio is 99.9 : 0.1.

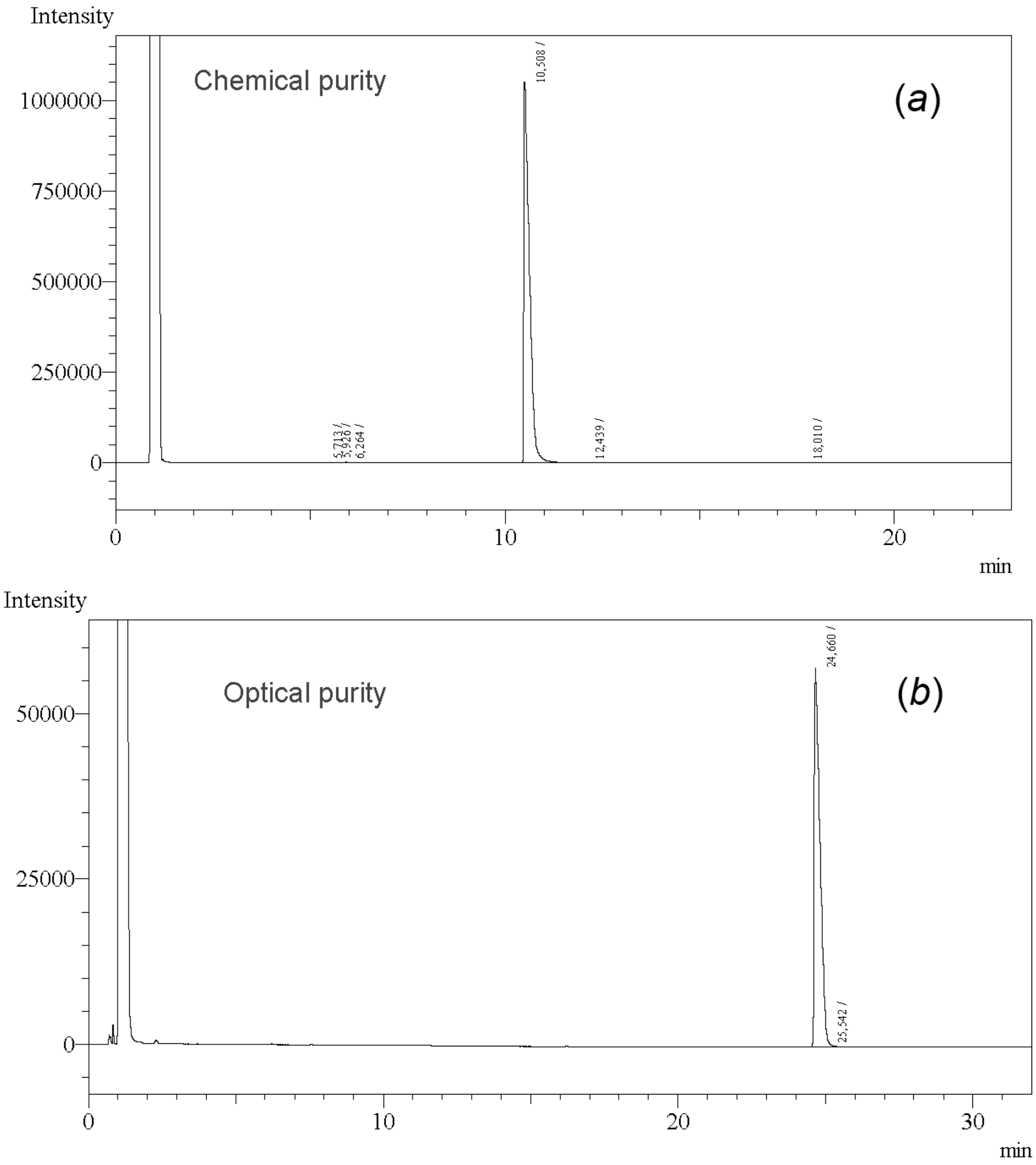

Figure S11. GC measurements of (*a*) chemical and (*b*) optical purity for (*S*)-MPD. The chemical purity is 99.7%; the enantiomeric ratio is 99.9 : 0.1.

Table S5 Calculated energies of the conformers of (*R*)-MPD in vacuum.

Conformer	E_{MP2} (Ha/mol)*	ΔE (kJ/mol)	ψ_1 (°)**	ψ_2 (°)**
**1a**	-386.5258854**	0.00	177.1	172.6
**3a**	-386.5215207	12.41	55.1	157.9
**2a**	-386.5209688	13.86	315.4	179.9
**3c**	-386.5207414	14.46	86.7	304.8
**3b**	-386.5196200	17.40	49.7	65.2
**1b**	-386.5188109	19.53	179.7	100.7
**2b**	-386.5174131	23.19	277.2	64.6
**1c**	-386.5173151	23.45	186.1	308.1
**2c**	-386.5156697	27.77	309.8	306.3

* Energy calculated using the second-order Møller-Plesset perturbation (MP2) method (Møller & Plesset, 1934).

** Torsion angles of the final structure.

Table S6. Root-Mean-Square Deviation between Equivalent C_α Atoms of Lysozyme

	**4B49** (no)	**4B4E** (*R*)	**4B4I** (*S*)	**4B4J** (*RS*)	1DPW (*RS*)	3B72 (*RS*)
**4B49** (no)		0.271	0.226	0.329	0.342	0.356
**4B4E** (*R*)	0.271		0.090	0.189	0.278	0.255
**4B4I** (*S*)	0.226	0.090		0.188	0.272	0.248
**4B4J** (*RS*)	0.329	0.189	0.188		0.219	0.176
1DPW (*RS*)	0.342	0.278	0.272	0.219		0.171
3B72 (*RS*)	0.356	0.255	0.248	0.176	0.171	

The first column lists the PDB code and MPD enantiomer(s) used to crystallize the protein. The colors are used to classify the structures by their similarity. The values (given in angstroms) were calculated using the SUPERPOSE algorithm in CCP4 (Winn *et al.*, 2011) by matching the C_α atoms of residues 1 to 129 for each pair of proteins. For residues with two or more conformations, the first (labeled A in the PDB file) was chosen. The most similar structures are highlighted in yellow (deviation < 0.1 Å), followed by those in green (0.1 Å < deviation < 0.2 Å) and those with no highlight (deviation > 0.2 Å). The four PDB codes in bold represent structures determined in this work. For comparison, we also include the structures determined by Weiss *et al.* [1DPW (Weiss *et al.*, 2000)] and Michaux *et al.* [3B72 (Michaux *et al.*, 2008)].

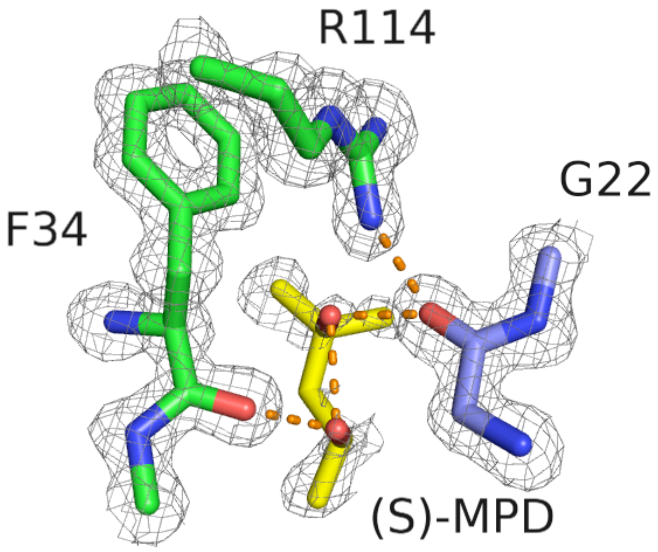

Figure S12. The crystal contact site near F34. The figure depicts the same site as Fig. 6*b* in the main text, except that the $2F_o$-F_c density is shown as a gray mesh contoured at 1.0σ (instead of 1.5 σ) to show more of the electron density around the (*S*)-MPD molecule.

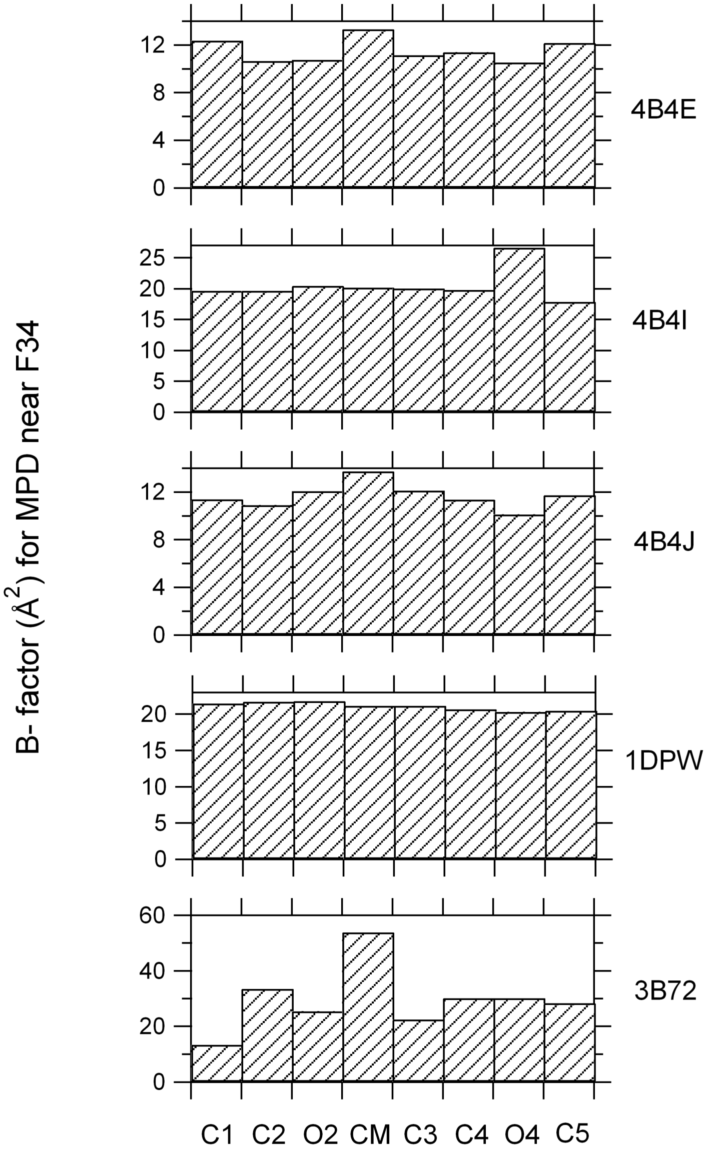

Figure S13. The B-factor for the MPD molecules at the F34 site. The nomenclature for the individual atoms follows that of Fig. 1 in the main text. The first four PDB codes represent structures determined in this work. The last two are for the structures determined by Weiss *et al.* [1DPW (Weiss *et al.*, 2000)] and Michaux *et al.* [3B72 (Michaux *et al.*, 2008)].

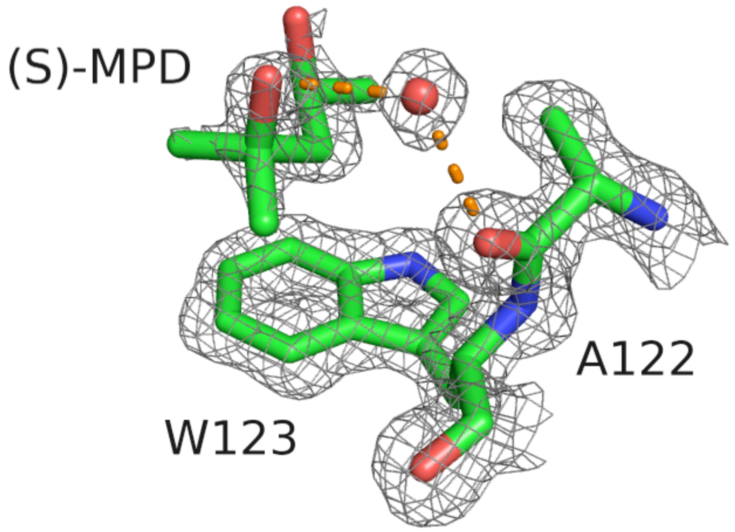

Figure S14. The interaction site near W123. The figure depicts the same site as Fig. 8 in the main text, except that the *$2F_o$-F_c* density is shown as a gray mesh contoured at 1.0σ (instead of 1.5 σ) to show more of the electron density around the (*S*)-MPD molecule.

Table S7. Comparison of crystal quality.

MPD additive	Maximum resolution (Å)	Maximum resolution (Å) at I/σI = 4.5	Wilson B factor (Å^2)	Mosaicity (°)
(*R*)-MPD [4B4E]	1.00	1.03	7.9	0.26
(*R*)-MPD	1.10	1.16	8.8	0.32
none [4B49]	1.15	1.18	10.0	0.32
none	1.22	1.27	11.0	0.43
(*S*)-MPD [4B4I]	1.20	1.24	12.3	0.46
(*S*)-MPD	1.25	1.27	12.5	0.59
(*RS*)-MPD [4B4J]	1.25	1.23	10.8	0.43
(*RS*)-MPD	1.10	1.13	8.6	0.45

For each precipitant the data from two different crystals is shown. The results corresponding to the four crystal structures discussed in the text are indicated by the PDB IDs in square brackets.

Supplementary References

Ewing, F. L., Forsythe, E. L., van der Woerd, M. & Pusey, M. L. (1996). *J. Cryst. Growth* **160**, 389-397.

Lomakin, A., Teplow, D. B. & Benedek, G. B. (2005). Vol. 299*, Methods in Molecular Biology. Amyloid Proteins: Methods and Protocols*, edited by E. M. Sigurdsson, pp. 153-173. Totowa, NJ: Humana Press Inc.

Michaux, C., Pouyez, J., Wouters, J. & Prive, G. G. (2008). *Bmc Struct Biol* **8**.

Møller, C. & Plesset, M. S. (1934). *Physical Review* **46**, 618-622.

Parmar, A. S., Gottschall, P. E. & Muschol, M. (2007). *Biophys. Chem.* **129**, 224-234.

Press, W. H., Teukolsky, S. A., Vetterling, W. T. & Flannery, B. P. (1992). *Numerical Recipes in C: The Art of Scientific Computing*, 2nd ed. Cambridge, UK: Cambridge University Press.

Weiss, M. S., Palm, G. J. & Hilgenfeld, R. (2000). *Acta Crystallogr D* **56**, 952-958.

Winn, M. D., Ballard, C. C., Cowtan, K. D., Dodson, E. J., Emsley, P., Evans, P. R., Keegan, R. M., Krissinel, E. B., Leslie, A. G. W., McCoy, A., McNicholas, S. J., Murshudov, G. N., Pannu, N. S., Potterton, E. A., Powell, H. R., Read, R. J., Vagin, A. & Wilson, K. S. (2011). *Acta Crystallogr D* **67**, 235-242.
